# Photochemical Modeling of Wintertime HO_*x*_ Sources and Sinks in a Subarctic Urban Location

**DOI:** 10.1021/acsestair.6c00254

**Published:** 2026-09-12

**Authors:** Alicia Hoffman, Rachel L. James, Dwayne E. Heard, Lisa Whalley, Daniel Stone, Samuel Seldon, Jochen Stutz, Jonas Kuhn, Sol Cooperdock, Brice Temime-Roussel, Amna Ijaz, Barbara D’Anna, Kathy S. Law, Slimane Bekki, Fangzhou Guo, James Flynn, James M. St Clair, Meeta Cesler-Maloney, William R. Simpson, Steve R. Arnold

**Affiliations:** † Institute for Climate and Atmospheric Science, School of Earth & Environment, 4468 University of Leeds, Leeds LS2 9JT, U.K.; ‡ School of Chemistry, 5292 University of Leeds, Leeds LS2 9JT, U.K.; § National Centre for Atmospheric Sciences, University of Leeds, Leeds LS2 9JT, U.K.; ∥ UCLA Atmospheric & Oceanic Sciences, Los Angeles, California 90095, United States; ⊥ Woodwell Climate Research Center, Falmouth, Massachusetts 02540, United States; # Aix-Marseille Université, CNRS, LCE, Marseille 13331 cedex 3, France; ∇ LATMOS/IPSL, 8783 Sorbonne Université, UVSQ, CNRS, Paris 75252, France; ○ Earth & Atmospheric Sciences, University of Houston, Houston, Texas 77204, United States; ◆ GESTAR-II, University of Maryland Baltimore County, Baltimore, Maryland 21250, United States; ¶ Geophysical Institute and Department of Chemistry and Biochemistry, 128791 University of Alaska Fairbanks, Fairbanks, Alaska 99775, United States

**Keywords:** Arctic, air pollution, OH radical, HO_*x*_ budget, box model

## Abstract

The hydroxyl radical (OH) is a key atmospheric oxidant that is important for regulating pollutant concentrations and particle formation and composition. The globally dominant formation mechanism of OH is ozone photolysis. However, the low-light conditions in the wintertime Arctic may limit OH formation through this pathway, and there is a lack of observations available to explain oxidant chemistry in polar locations. We use the Dynamically Simple Model of Atmospheric Chemical Complexity (DSMACC) 0D model to assess processes controlling the abundances of atmospheric oxidants in a subarctic city. To constrain the model, we use measurements made during the Alaskan Layered Pollution and Chemical Analysis (ALPACA) field campaign, which took place in Fairbanks, Alaska, USA, during the winter of 2022. HONO photolysis to form OH is the major chemical source of HO_*x*_ in the polluted, low-light environment of wintertime Fairbanks, and formaldehyde is an important precursor for HO_2_. We find that the HO_*x*_ budget is distinct between a polluted, strongly stable inversion event and a clean, weakly stable period because of the different meteorology and pollution levels regulating peroxynitric acid (PNA, HO_2_NO_2_) cycling. Our results improve our understanding of the unique atmospheric pollution chemistry under cold and low-light conditions.

## Graphical abstract

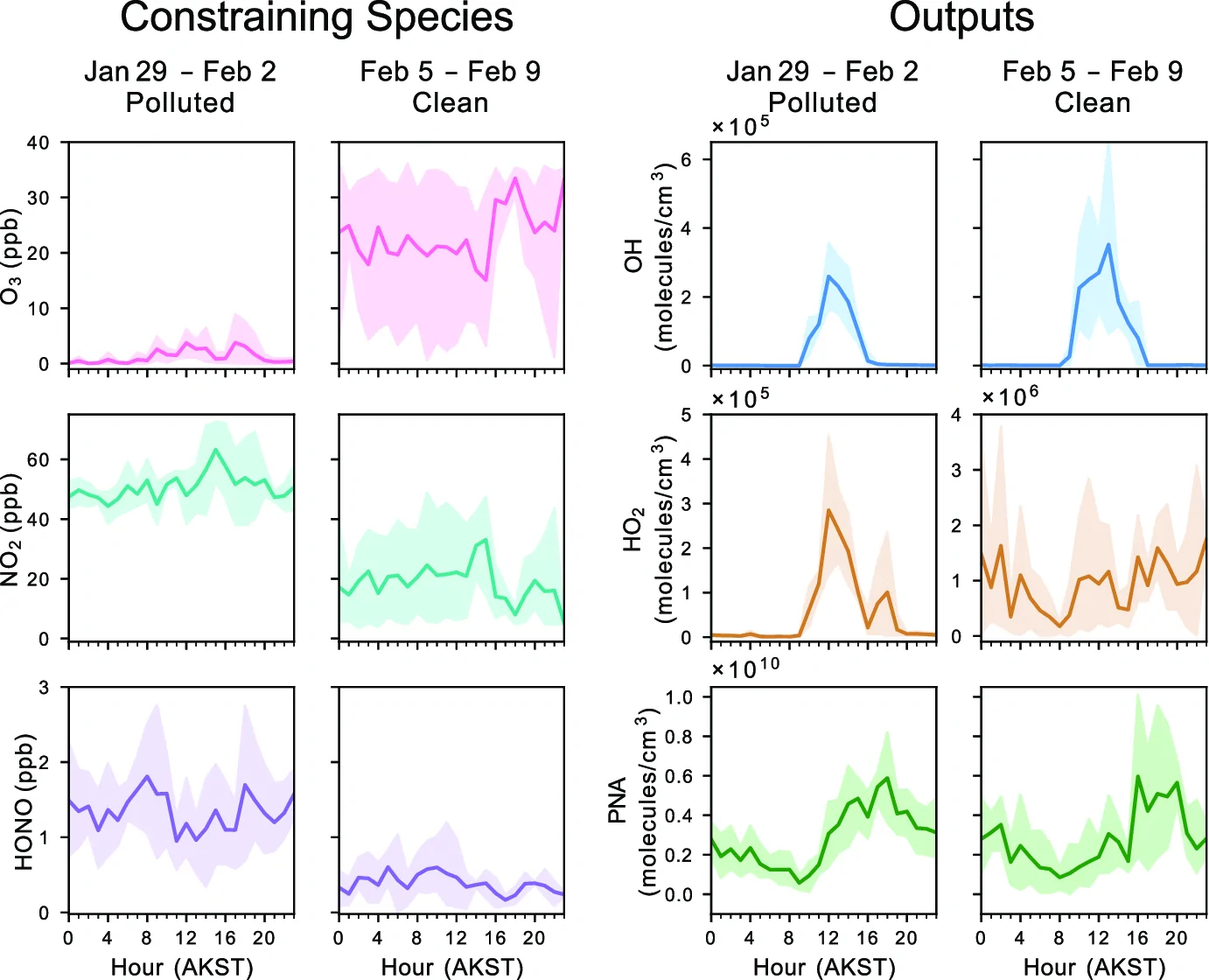

## 1 Introduction

Atmospheric oxidants are key players in chemical reactions that result in the transformation and loss of atmospheric pollutants and the formation of secondary pollutants. These oxidants include the hydroxyl radical (OH), ozone (O_3_), and nitrate radical (NO_3_). The reaction with OH is the dominant loss process for most reactive gases in the atmosphere.^1,2^ As such, OH regulates the conversion of primary emissions to secondary species,^3^ influences particulate matter concentrations through the formation and growth of secondary aerosols,^4^ and on a large scale determines the lifetime of the greenhouse gas methane.^5,6^

In polar regions, the cold temperatures, low solar insolation, and high solar zenith angle during winter limit the formation of OH,^7,8^ resulting in prolonged lifetimes of reactive pollutants relative to typical low- to midlatitude conditions.^9^ Production of OH through the globally dominant formation mechanism in [Disp-formula eq1] and 2 is expected to be low in polar winter due to limited solar insolation at short wavelengths, suppressing the photolysis of O_3_ in [Disp-formula eq1], and cold temperatures limiting water vapor concentrations (H_2_O), suppressing [Disp-formula eq2].^10–13^ In several winter environments, 3 has been found to dominate OH production because there is more availability of light at the longer wavelengths needed for HONO photolysis than at the shorter wavelengths for ozone photolysis.^14,15^ However, the dominance of HONO photolysis has not been seen in all wintertime studies of OH.^10^ In addition, polluted conditions in some wintertime locations affect the formation of OH via [Disp-formula eq4] and the availability of the HO_*x*_ reservoir species peroxynitric acid (PNA, HO_2_NO_2_) in [Disp-formula eq5], although these results have not been found in all locations.^11,16,17^ There is a lack of field measurements to corroborate the expected chemical regimes in Arctic locations,^18^ and modeling of wintertime OH has revealed uncertainties in chemical mechanisms such as the role of heterogeneous chemistry and missing reactivity.^17,19–22^

R1 O3+hν(≤320nm)→O(D1)+O2

R2 O(D1)+H2O→2OH

R3 HONO+hν(400nm)→OH+NO

R4 $${\mathrm{HO}}_{2}+\mathrm{NO}\to \mathrm{OH}+{\mathrm{NO}}_{2}$$

R5 $${\mathrm{NO}}_{2}+{\mathrm{HO}}_{2}↔{\mathrm{HO}}_{2}{\mathrm{NO}}_{2}$$

The distribution of wintertime surface observations of either OH concentration or reactivity has a marked focus on northern-hemisphere, midlatitude locations (Figure 1). Aircraft flight campaigns such as TOPSE or ATom^23,24^ have measured wintertime concentrations aloft rather than at the surface; a comprehensive comparison of OH measurements made during flight campaigns is given in Miller and Brune.^25^ Several recent surface-based field studies have focused on Beijing and the North China Plain due to their intense wintertime haze events.^10,11,16^ There are also winter measurements in urban areas such as New York City, Tokyo, and Birmingham, UK,^14,15,21,26–28^ as well as rural areas.^17,29–32^ Some studies have demonstrated differences between winter and summer oxidant concentrations and VOC lifetimes in given locations.^14,27,30,33,34^ However, none of these studies capture the extreme cold and dark conditions experienced in polar regions during winter.

**Figure 1:**
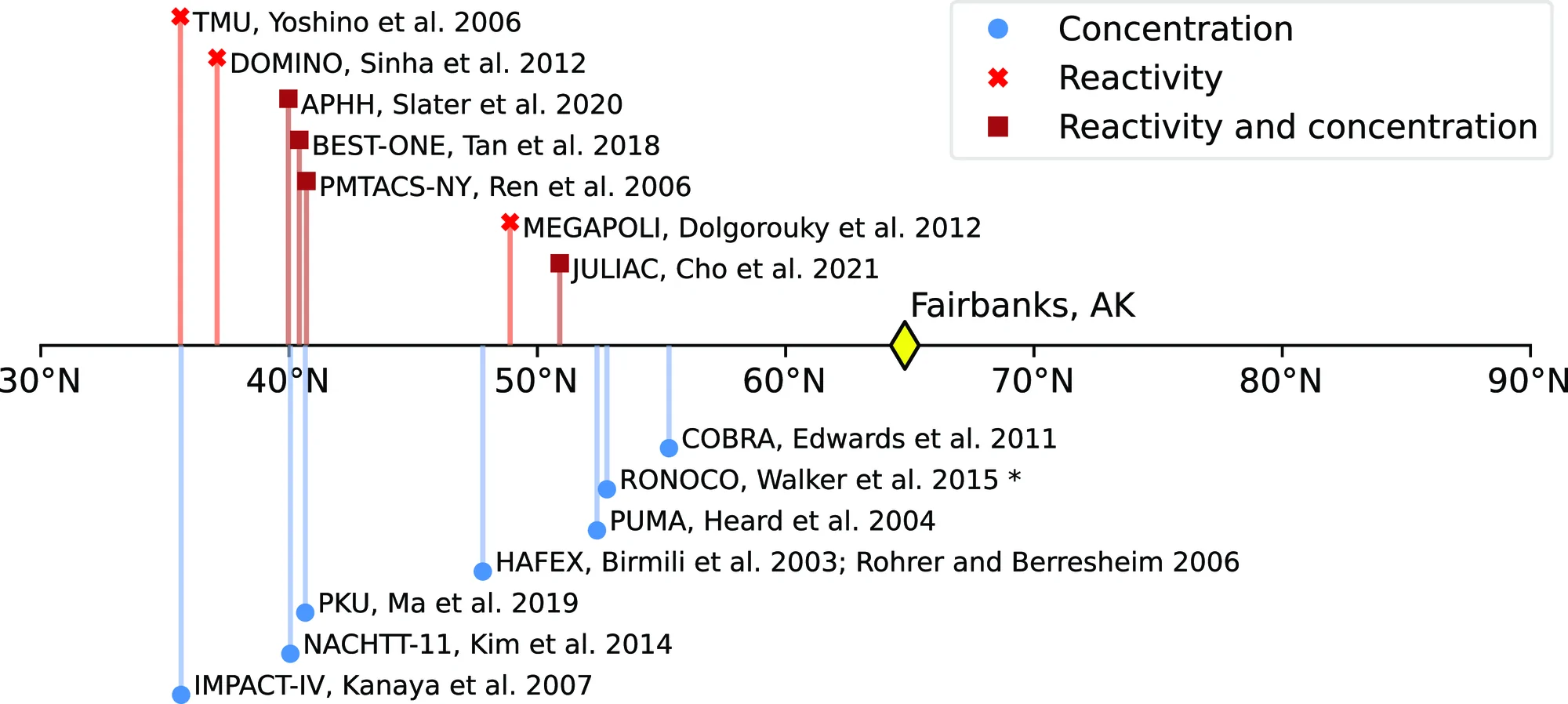
Latitudes of field studies that included measurements of surface level atmospheric OH concentration (blue circles), reactivity (red crosses), or both (brown squares). The name of the field campaign or the measurement location is listed with the reference for the OH measurements. Only campaigns that included surface wintertime (DJF) measurements are included. We found no studies in the southern hemisphere of wintertime (JJA) measurements and therefore did not include this region in the *x*-axis. Only campaigns that measured reactivity were included, and campaigns that calculated reactivity based on measured VOC concentrations were not. The RONOCO campaign, marked with *, intended to measure OH but concentrations were lower than the limit of detection and Walker et al.^31^ gives an upper limit to the OH concentration for this campaign.

The Alaskan Layered Pollution and Chemical Analysis (ALPACA) 2022 field project took place from January to March 2022 in Fairbanks, Alaska, USA, with the goal of better understanding atmospheric chemistry during high-latitude winter in urban environments.^35^ Fairbanks is located at 64.8 °N and experiences poor air quality each winter.^36^ Strong and persistent surface-based temperature inversions trap local emissions and often lead to PM_2.5_ concentrations that exceed the US Environmental Protection Agency (EPA) ambient air quality standards.^37,38^ There are uncertainties surrounding the chemistry that occurs during these pollution events. Specific open research questions include: which oxidants are present under cold and dark conditions, what are their roles in SOA formation and sulfur oxidation, and how does reactive nitrogen chemistry affect oxidant concentrations? Some studies based on data from the ALPACA project have examined the aspects of oxidant chemistry in Fairbanks, e.g., Farrell et al.,^39^ but as of yet there have been no modeling studies of the chemistry controlling OH during the campaign.

Here, we examine oxidant chemistry in Fairbanks during the winter, with a primary focus on the OH and HO_2_ (HO_*x*_) budgets using a combination of chemical modeling and field measurements from ALPACA 2022. We estimate concentrations of HO_*x*_ during ALPACA using an established box modeling framework. We use the Dynamically Simple Model of Atmospheric Chemical Complexity (DSMACC), a zero-dimensional model,^40,41^ constrained with measurements made during ALPACA 2022 to simulate near-surface photochemistry during the campaign period. The goal of this work is to identify key atmospheric processes that affect the oxidation capacity and secondary air pollution formation in urban polar winter conditions.

## 2 Materials and Methods

### 2.1 Model Setup

To simulate near-surface photochemistry in Fairbanks, we used the Dynamically Simple Model of Atmospheric Chemical Complexity (DSMACC).^40,41^ We used the Master Chemical Mechanism (MCM) v3.3.1,^42,43^ available at https://www.mcm.york.ac.uk (last access: Nov 17, 2025) processed with Kinetic PreProcessor (KPP) v2.2.3.^44^ Clear-sky photolysis rates were calculated using Tropospheric Ultraviolet and Visible (TUV) Radiation Model v4.2;^45^ the measured *J*_O^1^D_ and J_NO_2__ from the ALPACA campaign^46^ were used to scale other clear-sky photolysis rates to account for cloud cover.

We constrained the model by using measurements made during the ALPACA campaign. These measurements included available VOCs (listed in Table 1) measured by proton transfer reaction time-of-flight-mass spectrometry (PTR-ToF-MS);^47,48^ formaldehyde measured by COFFEE (compact formaldehyde fluorescence experiment) and Aeris (mid-infrared absorption) instruments;^49,50^ NO, NO_2_, SO_2_, CO, and O_3_ by Thermo Scientific gas analyzers;^51^ and HONO concentration measured aloft (from 12 to 17 m) by long-path differential optical absorption spectroscopy (LP-DOAS).^52^ For each *m*/*z* detected by the PTR-ToF-MS, major VOCs contributing to the signal at the corresponding ionic formula to organic compounds were assigned based on previous references for VOC emission profiles associated with sources identified in Fairbanks in wintertime,^53^ thus reducing to the subset of representative compounds listed in Table 1. All concentrations except HONO were measured at the same ALPACA supersite in downtown Fairbanks, while the HONO measurements were made nearby.^35^ We resampled observations at a frequency of 20 min to correspond to the HONO measurements, which had the coarsest temporal resolution. Model simulations were conducted for periods when valid measurement data of all inputs were available, giving a total of 593 modeled data points across the 40 day campaign. Figure 2 shows a time series of a subset of the input data used to constrain the box model. The gaps in the time series are due to one or more missing inputs for each 20 min of resampling interval. The resulting model output is consistent with this 20 min frequency, including gaps in the time series (i.e., the model does not interpolate between data points). The distributions of CO mixing ratios measured during the entire ALPACA campaign period and for the subset time series of data presented in Figure 2 agree well (see Supporting Information Figure S1). This implies that our modeling results are representative of the conditions sampled throughout the ALPACA campaign.

**Figure 2:**
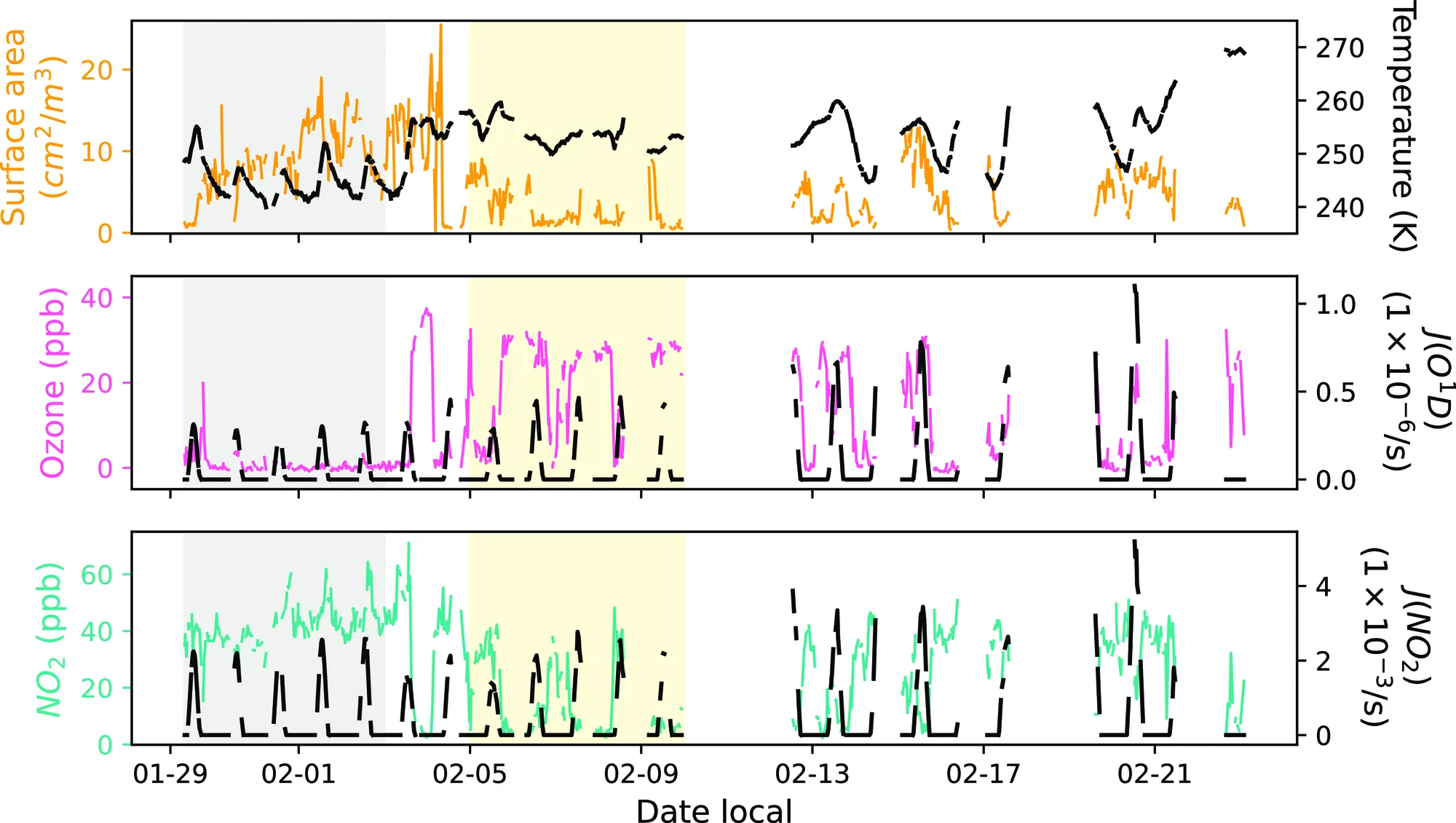
Time series of a subset of inputs used in the box model to constrain the simulations. Surface area (orange) and temperature (black) in the top row, ozone (pink) and *J*_*O* ^1^D_ (black) in the middle row, and NO_2_ (cyan) and J_NO_2__ (black) on the bottom row. The shading denotes the two time periods selected for case study analysis. Period 1 (Jan 29 through Feb 02) in gray shading is a polluted period, during which NO_2_ and particle pollution were high but ozone was depleted. Period 2 (Feb 05 through Feb 09) in yellow is a clean period, when NO_2_ and particle pollution are low but ozone reaches ambient concentrations upward of 35–40 ppb. Sunlight increases throughout the campaign period, as represented by the increasing and broadening J_O^1^D_ and J_NO_2__.

**Table 1:** Average Concentrations of VOCs Included as Constraining Species

		percentile	
gas	mean (ppb)	10th (ppb)	90th (ppb)
constrained in all simulations			
formaldehyde^a^	3.972	0.983	9.08
methanol	5.394	1.104	10.81
acetaldehyde	1.721	0.627	3.09
formic acid	0.915	0.580	1.30
ethanol	3.827	1.025	6.51
acetone	1.249	0.548	2.10
acetic acid	1.973	0.445	3.70
MVK + MACR^b^	0.171	0.030	0.37
butanone	0.179	0.098	0.28
benzene	0.952	0.167	2.17
2,3-butanedione	0.180	0.050	0.32
toluene	2.255	0.239	5.42
phenol	0.086	0.007	0.18
acetic anhydride	0.027	0.009	0.05
styrene	0.074	0.007	0.17
2,5-hexanedione	0.016	0.003	0.03
benzoic acid	0.034	0.003	0.08
constrained in sensitivity analyses			
isoprene^c^	0.414	0.061	0.87
ethane^d^	2.591	1.081	4.54
ethene^d^	15.383	3.234	31.73
propane^d^	0.740	0.318	1.36
propene^d^	5.164	1.043	10.67
*n*-butane^d^	0.232	0.076	0.46
*i*-butane^d^	0.102	0.031	0.22

a Measured by COFFEE and Aeris, not by PTR-ToF-MS.

b PTR-ToF-MS signal reported as MVK + MACR, represented as only MVK in the model.

c The PTR-ToF-MS ion was not conclusively identified.

d Estimated concentration.

As shown in Figure 2, there are some significant gaps in the temporal coverage of the model inputs. We therefore chose two case study periods during which the temporal frequency of model inputs was high and conducted a detailed comparison of these two periods to demonstrate the variability of the HO*_*x*_* chemistry throughout the campaign. Period 1 (Jan 29 through Feb 02, gray-highlighted period in Figure 2) was in the middle of a strong surface-based inversion event, which resulted in high PM_2.5_ and NO*_*x*_* concentrations, and we refer to this period as the polluted period.^35,54^ We considered Period 2 (Feb 05 through Feb 09, yellow-highlighted period in Figure 2) a clean event. During this period, there was more advection and mixing from aloft and only weakly stable conditions.^54^ The two case study periods occurred during the first half of the ALPACA campaign, coinciding with low sunlight levels and cold temperatures. However, the average temperature during Period 2 (clean period) was about 10 K warmer than that during Period 1 (polluted period), 245 K compared to 254 K.

The DSMACC model does not explicitly simulate nonchemical loss of intermediate species via physical processes such as deposition, mixing, and advection. To prevent an unrealistic accumulation of the species not constrained by measurements, we add a first-order loss term (*k*_phys_) for all species with a rate coefficient of 1.16 × 10^–5^ *s* ^–1^ equivalent to a lifetime of approximately 24 h. This is based on the work of Stone et al.,^41^ which showed that HO_*x*_ concentrations were not significantly impacted by changes to deposition lifetime in the model. We address this assumption by testing the sensitivity of the model to a range of *k*_phys_ values. As the MCM only comprises gas-phase chemical reactions, we introduce heterogeneous loss for OH and HO_2_ onto aerosol surfaces as first-order loss terms (eq 6) where *k*_het_ is the heterogeneous loss first-order rate coefficient, *SA* is the effective surface area per unit volume (cm^2^/cm^3^), which we constrained in the model with measurements made by a scanning mobility particle sizer (SMPS),^48^ and γ_*i*_ is the unitless uptake coefficient of gas *i*. The mean molecular speed for each gas *c*_*i*_ is shown in eq 7, where *R* = 8.31451 J·mol^–1^·K^–1^ is the ideal gas constant, *T* is the temperature in Kelvin, and *M*_w_ is the molar mass of the gas. We include γ_HO_2__ = 0.1 and γ_OH_ = 1.0 as the default simulation values and test the sensitivity of the model to a range of uptake coefficient values.

1 $$k_{\mathrm{het}}=\frac{c_{i}\mathrm{SA}\gamma_{i}}{4}$$

2 $$c=\sqrt{\frac{8\mathrm{RT}}{\pi M_{w}}}$$

The relative humidity in the base simulation was set to 72% as an average of the available measurements from Fairbanks International Airport during the ALPACA project. The water vapor volume mixing ratio was calculated from the average relative humidity, observed pressure collected at the Fairbanks International Airport, and the observed temperature from the downtown ALPACA supersite. The pressure measurements were made on an hourly basis, so we resampled them to 20 min frequency using a linear interpolation to calculate water vapor volume mixing ratio. The mixing ratio of CH_4_ was set to a constant value of 2019 ppb based on the average of available *in situ* and flask observations from January through February made at the Barrow, Alaska, and Cold Bay, Alaska, NOAA Carbon Cycle Cooperative Global Air Sampling Network sites.^55^ We set H_2_ to a constant mixing ratio of 516 ppb, as the average of flask measurements made between January and February 2022 from the Barrow and Cold Bay sites.^56^ Broadband albedo was set to 0.7, based on a mean of albedo values calculated from observed *J*_NO_2__ radiation fluxes during the campaign (mean 0.66, minimum 0.54, and maximum 0.99).

### 2.2 Sensitivity Analysis

We conducted several sensitivity experiments to better understand the factors influencing our model. We tested the sensitivity of the model-simulated HO*_*x*_* to loss via physical processes (*k*_phys_), HONO concentration, relative humidity, uptake of HO_2_ and OH to particles (γ_HO_2__ and γ_OH_), and constrained VOC inputs. For all sensitivity analyses, we evaluated the change in midday (10 am to 2 pm) local Alaska Standard Time (AKST) OH and HO_2_ concentrations.

We adjusted *k*_phys_ for all species in two sensitivity experiments: decreasing *k*_phys_ so that the lifetime with respect to physical loss would be approximately 5 days and increasing *k*_phys_ so the lifetime would be approximately 1 h. We conducted a third sensitivity experiment in which we adjusted only the physical lifetime of PNA to 1 h, *k*_phys,PNA_ = 2.78 × 10^–4^ *s* ^–1^, to isolate the impact of PNA, and investigate how its potential accumulation or dispersal may affect the HO*_*x*_* budget.

We adjusted HONO concentration in two sensitivity tests by ±20% to evaluate the impact of uncertainty in the observed HONO. All measurements used in our modeling aside from HONO are made at the surface. The remote sensing measurements sample HONO concentrations from 12 to 17 m height above the surface. We expect that the degree to which the ambient HONO concentration in Fairbanks is well-mixed and representative between the surface and aloft will be sensitive to vertical stability and may differ between the polluted (stable) and cleaner (weakly stable) periods.

We conducted three sensitivity tests with RH set to 30, 60, and 90% in the calculation of the water vapor mixing ratio. These values cover the range of RHs measured at the Fairbanks International Airport. Relative humidity and pressure were used to calculate the water vapor mixing ratio. The pressure measurements we used were interpolated from an hourly frequency to 20 min to align with other ALPACA measurements. These three RH sensitivity tests allowed us to evaluate both the impact of interpolating the coarser resolution data and the impact of using a fixed average RH on model output, as the change in water vapor mixing ratio from changing RH was greater than the change introduced by adjusting pressure.

We tested the impact of removing OH uptake (γ_OH_ = 0) and decreasing γ_HO_2__ from 0.1 in the base simulation to 0.01 and 0.001. Recent laboratory studies have found that OH reactive uptake depends on a number of factors including particle composition, temperature, and relative humidity.^57–60^ Reactive uptake and heterogeneous oxidation by OH are considered important drivers of organic aerosol chemical aging.^59,61,62^ Because of the wide range of γ_*OH*_ values in the literature,^63,64^ we tested the sensitivity of our model using extremes of this range. There is also a wide range of γ_HO_2__ values in the literature from lab, modeling, and field studies, which show dependence on factors such as particle composition, pH, ambient temperature, and NO*_*x*_* concentration.^20,65–68^ A typical value used in models is γ_HO_2__ = 0.2 based on observations made and comparison with a model during the 1997 SONEX field campaign.^69,70^ However, later field measurements have found that γ_HO_2__ can vary widely even over a short period in one location.^71–74^ Zhou et al.^71^ recommended using different uptake values in models to be representative of the area of interest, but there is limited analysis of wintertime HO_2_ uptake in the Arctic. To account for the lack of available literature values, we tested γ_HO_2__ over 3 orders of magnitude.

In our default model setup, we used a limited number of VOCs to constrain the model based on available measurements. First, we assumed that the *C*_5_H_9_^+^ ion measured by PTR-ToF-MS was not isoprene based on the recent literature, suggesting that fragments of aldehydes and cycloalkanes from anthropogenic sources including fossil fuels and cooking can interfere with the detection of C_5_H_9_^+^ by PTR-MS.^75,76^ In Fairbanks, enhancement in the C_5_H_9_^+^ ion is associated with home heating oil and traffic.^53^ There is also likely a negligible contribution from biogenic isoprene in this winter urban environment. We therefore excluded this species as a constraint in the base model setup. As the C_5_H_9_^+^ fragment in the ALPACA measurements has not been conclusively attributed to isoprene or another VOC, we performed a second simulation with an identical setup and inputs to our base simulation but with the model constrained to C_5_H_9_^+^ concentration under the assumption that it was isoprene. Because isoprene has higher reactivity with OH than other species that produce the C_5_H_9_^+^ fragment such as octanal and nonanal,^75,77^ this constrained simulation (called isoprene constrained, IC) and the base simulation represent extremes of the potential impact of the C_5_H_9_^+^-based constraint on the HO_*x*_ budget and concentrations.

In addition, *C*_2_–*C*_4_ alkanes and alkenes were not measured by PTR-ToF-MS and therefore we did not include these species in our base model simulation. We conducted a sensitivity test with estimated ethane, ethene, propane, propene, *i*-butane, and *n*-butane concentrations (Table 1) added to the model as constraining species. We estimated the *C*_2_–*C*_4_ alkane and alkene concentrations using emission ratios (ERs) from the major sources of VOC emissions in Fairbanks. Our calculation of VOC concentrations using these ERs is intended to allow a simple sensitivity of model HO*_*x*_* to potential sources of additional VOCs in Fairbanks. We focused on the primary emission sources of biomass burning (i.e., wood combustion), heating oil, and traffic, which were identified by PMF analysis as being important factors in Fairbanks by Ketcherside et al.^53^ We use ERs from Permar et al.^78^ for the long-lived biomass burning factor. The published ERs are in ppb VOC per ppm CO, which we converted to ppb VOC per ppb formic acid, as about 90% of the measured formic acid in Fairbanks is from biomass burning.^53^ For traffic emissions, we used measured emission factors from Gordon et al.,^79^ which are reported in mg/kg of fuel. We convert these emission factors to ERs of ppb VOC per ppb NO*_*x*_*, as about 55% of NO*_*x*_* in Fairbanks was from vehicles. Emissions of VOCs from vehicles depends on the engine type, fuel type, driving conditions, and vehicle maintenance^80–83^ and measured emissions has varied between locations and over time.^83–85^ In general, *C*_2_–*C*_4_ alkanes and alkenes are a small contribution of vehicle exhaust, although some species are a larger fraction of fuel evaporation, which we do not consider in our calculations.^80,86,87^ Finally, for fuel oil, we used measurements from a residential oil burner study reported in mg/kg fuel.^88^ We converted these emission factors to ERs of ppb VOC per ppb benzene, as heating oil was a major source (approximately 35%) of benzene during the ALPACA project.^53^ For each source, we weight the representative species concentration *C*_rep_ (i.e., formic acid, NO*_*x*_*, and benzene) by the relative contribution of the source they represent as determined by Ketcherside et al.^53^ (PMFfraction) to calculate the species concentrations *C*_*i*_ as shown in eq 8.

3 $$C_{i}=C_{\mathrm{rep}}\times \mathrm{PMFfraction}\times ER_{i}$$

Adding the new VOCs to our constrained species required that the mechanism be updated for all MCM reactions that included these species and their intermediates. We tested the new mechanism with the base set of constrained concentrations, excluding the six new VOCs and isoprene, to isolate the impact of adding further reactions to the mechanism. There was an average of −0.03% difference in both OH and HO_2_ concentrations between the updated mechanism simulation and the base simulation, both run with the same constraining species. Thus, just adding reactions with the new VOCs without constraining these species had limited impact on the model.

## 3 Results and Discussion

### 3.1 HO_*x*_ Concentrations in Fairbanks

Concentrations of HO_*x*_ and PNA throughout the ALPACA campaign calculated by DSMACC are shown in Figure 3. Concentrations of OH and HO_2_ are low in January and start to increase during early- to mid-February as sunlight increases. There were more than four additional hours of sunlight by the end of the ALPACA campaign compared to the beginning.^35^ OH has a clear dependence on the time of day, with a peak in average diel concentration of 3.7 × 10^5^ cm^–3^ at 1 pm and close to zero between 5 pm and 8 am. The HO_2_ concentration is higher through much of the day compared to OH but shows little variation with sunlight. Peak average diel HO_2_ concentration is 1.4 × 10^6^ cm^–3^ at 3 pm, while the concentration at noon is half of this. During some periods of the campaign, such as from Feb 14 to Feb 24, HO_2_ is maximum during the day, in line with OH. During other periods, such as between Feb 04 and Feb 09, there are large spikes of HO_2_ during the night. This results in a large range in diel HO_2_ concentration, with the exception of 8 am local time, when day-to-day variability in HO_2_ is minimal. This dip in HO_2_ variability is due to depletion overnight prior to the beginning of photolysis-driven production and will be explored further in Section 3.1.1. The total HO*_*x*_* concentration follows a similar pattern as HO_2_, but with a small increase in mean concentration at 1 pm relative to HO_2_ because of the peak in OH concentration at this time. The concentration of PNA peaks in the early evening and decreases to a minimum in the morning. This diel trend is quite clear during the polluted period from Jan 29 to Feb 03 in the time series, but the diel cycle of PNA concentrations becomes more variable later in the time series. From Feb 05 onward, PNA concentration tracks with HO_2_, potentially contributing to the elevated concentrations of HO_2_ at night. This relationship will be explored more in the budget analysis in Section 3.2.

**Figure 3:**
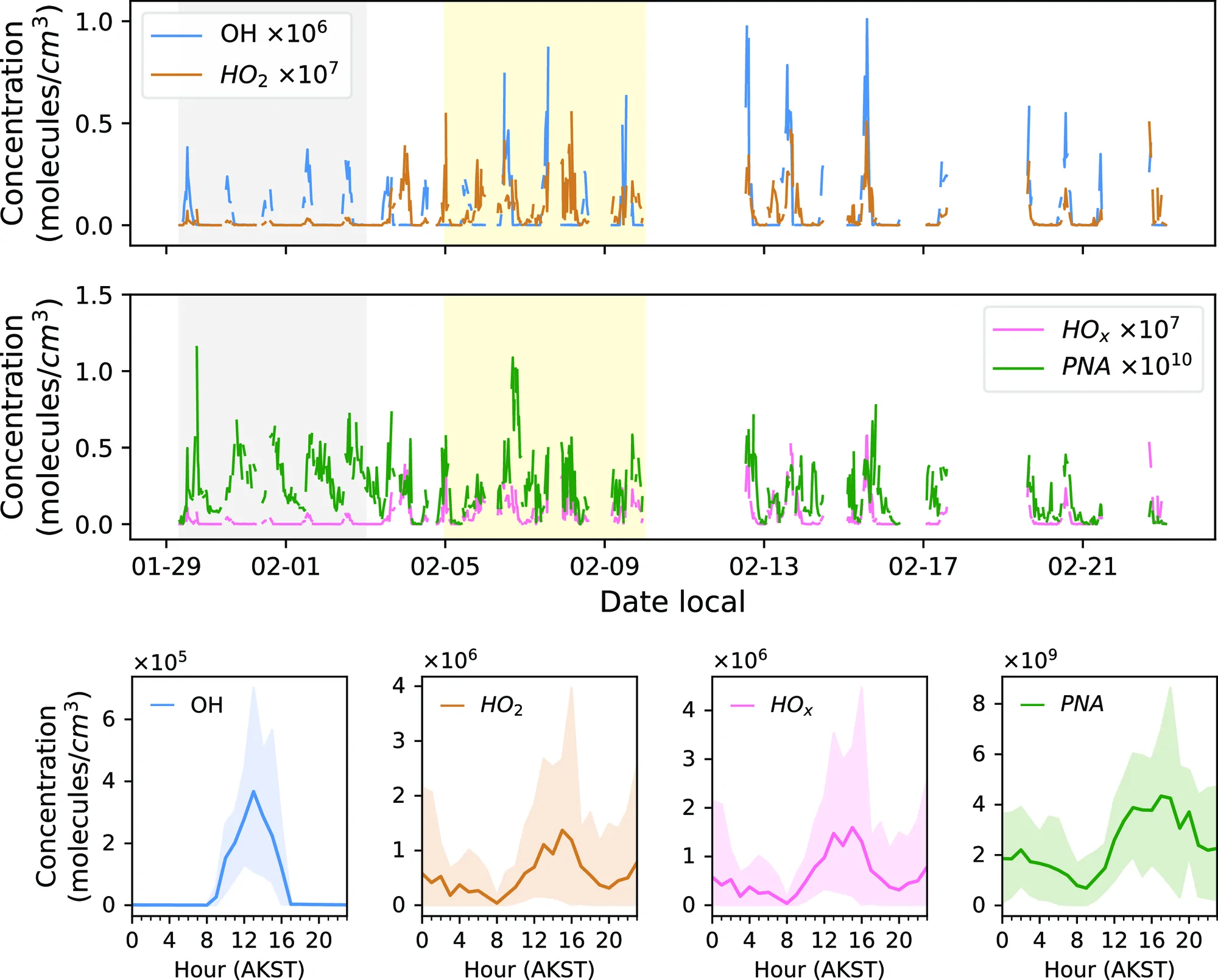
Time series of OH and HO_2_ (top) and HO*_*x*_* and peroxynitric acid (PNA) (middle) during the ALPACA campaign. Mean diels are plotted along the bottom, with shading to show the 10th and 90th percentile of concentration each hour. The shaded regions in the upper panels denote the time range for the case study periods: Period 1 in gray and Period 2 in yellow.

Our modeled concentrations of HO*_*x*_* in Fairbanks are lower than measurements made in most urban locations in winter. Measured wintertime concentrations of OH were on the order of 2 × 10^6^ cm^–3^ or greater in Beijing,^10,11,16^ New York City,^14^ Tokyo,^33^ Boulder, CO,^15^ and Birmingham, UK.^27^ The two most northerly campaigns shown in Figure 1 report OH concentrations of a similar magnitude as calculated in our model. During the RONOCO campaign in the UK, nocturnal wintertime OH concentrations were below the instrument detection limit, and the estimated maximum OH concentration at night was 6.4 × 10^5^ cm^–3^.^31^ During the COBRA campaign in rural Canada, peak noon concentrations were 1.16 × 10^6^ cm^–3^ but were below the detection limit (6.4 × 10^5^ cm^–3^) for much of the rest of the day.^17^ Wintertime HO_2_ concentrations ranging from 0.4 × 10^8^ to 4.5 × 10^8^ cm^–3^ were made in Beijing, Tokyo, New York City, and Birmingham,^10,11,14,16,27,33^ which are 1–2 orders of magnitude greater than those calculated in our model. HO_2_ concentration in the COBRA campaign was also greater than that calculated here, peaking at 1.42 × 10^8^ cm^–3^ at noon, closer to the other urban measurements. We explore the drivers of the estimated HO_*x*_ concentrations simulated in Fairbanks in our model budget analysis.

#### 3.1.1 Comparison of Clean and Polluted Periods

We compared the OH, HO_2_, and PNA concentrations during two different periods of the campaign to better understand the influence of pollution abundance on the HO_*x*_ chemistry in Fairbanks. The diels in Figure 4 demonstrate that concentrations were distinctly different between the two periods. During the polluted period, the level of NO_2_ is greater than 40 ppb for the entire day. The build-up of NO_2_ coincides with near-zero O_3_ concentration as ambient O_3_ is titrated with NO. This chemistry during the polluted event is driven by stable atmospheric conditions that trap local NO*_*x*_* emissions but prevent mixing with O_3_-rich air from outside Fairbanks.^35,54^ In comparison, during the clean event, weakly stable meteorology allows for mixing- and transport-driven dispersion of local emissions, reducing the NO*_*x*_* concentrations. Thus, while we describe the impact of different chemical regimes on HO*_*x*_*, these regimes are influenced by varying meteorological conditions across the campaign period.

**Figure 4:**
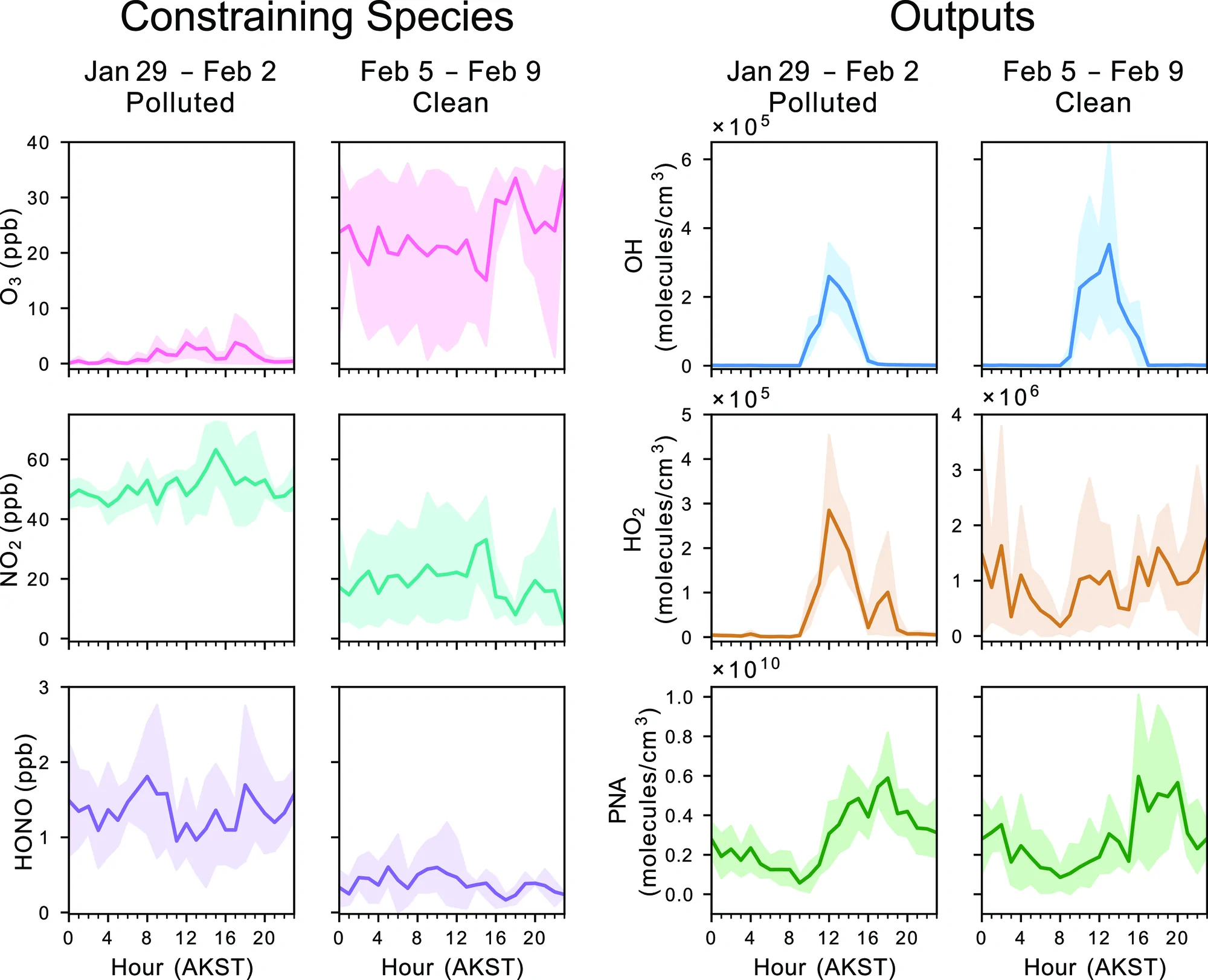
Diel concentrations of selected species for the clean (columns 2 and 4) and polluted (columns 1 and 3) periods. Concentrations for constraining inputs are shown in the left two columns: ozone (pink, top row), NO_2_ (cyan, middle row), and HONO (purple, bottom row). Model outputs are shown in the right two columns: OH (blue, top row), HO_2_ (brown, middle row), and PNA (green, bottom row). Species are plotted on the same *y*-axis scale between the polluted and clean periods except for HO_2_ because the concentration during the polluted period is an order of magnitude smaller than during the clean period. Shading for all species represents the 10th and 90th percentiles.

HO_*x*_ concentrations are lower on average during the polluted period compared to the clean period, and HO_2_ concentration in the clean period follows a similar diel cycle as OH with a peak in concentration around midday. During the clean period, O_3_ concentration is high, sometimes reaching 35–40 ppb, and is consistent with concentrations in ambient air masses outside the Fairbanks urban area.^35^ The NO_2_ concentration is much lower during the clean period than during the polluted period. The HO_2_ concentration does not show a strong daytime peak or dependence on the time of day in the clean period. In both clean and polluted periods, the HO_2_ concentration is at a minimum at 8 am, which was reflected in the small concentration variability in the campaign average diel in Figure 3. The OH diel profiles are broadly similar between the two periods, despite differences in HO_2_. Studies in Beijing have also reported a similar OH behavior during haze versus nonhaze events.^10,16^ However, during these studies, HO_2_ concentrations were also similar during haze and nonhaze events,^11,16^ indicating that there may be different pollution sources and chemistry occurring in Beijing haze events compared to Fairbanks pollution events. The PNA diels are also broadly similar between the two periods despite the differences in NO_2_ and HO_2_ concentrations. The stark differences between HO*_*x*_* and PNA concentrations between the clean and polluted periods hint at different chemical regimes, which will be explored further in budget analysis.

### 3.2 HO*_*x*_* Budget

The major chemical production and loss reactions in the box model for the campaign average are shown in the rate of production and loss analyses (ROPAs) in Figure 5. Production of OH in Fairbanks is dominated by HONO photolysis, which peaks at around midday. The reaction of HO_2_ with NO is the second-largest source of OH, and this reaction continues to produce OH after sunset. The loss of OH is dominated by the reaction with NO_2_ to form HNO_3_. There is also some recycling of NO and OH to reform HONO, although loss by this mechanism does not balance with the production of OH from HONO. Reactions with acetaldehyde and CO are also sinks of OH during the day. Acetaldehyde during the ALPACA campaign is mainly produced from wood burning and heating oil combustion.^53^

**Figure 5:**
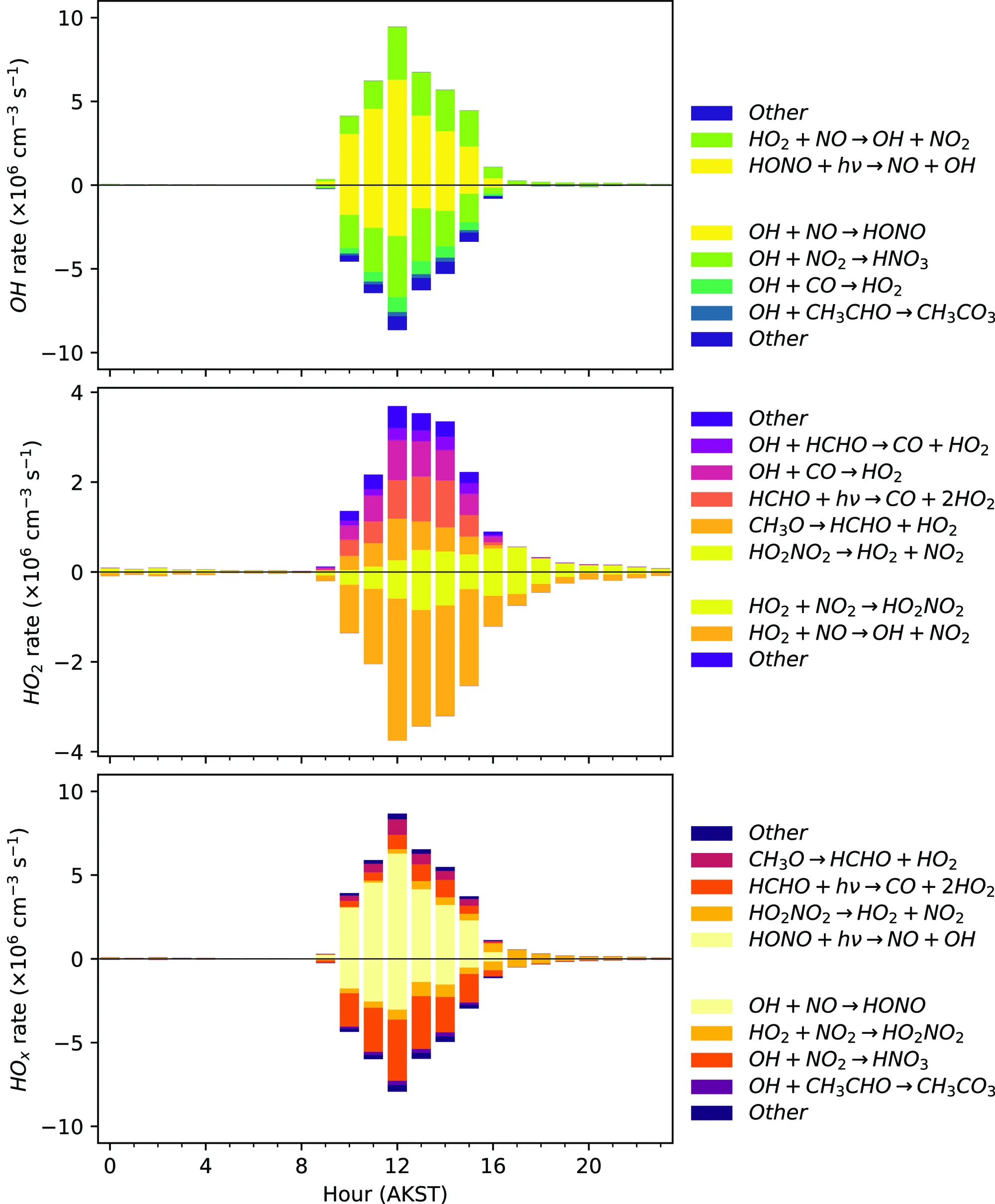
Rate of production and loss analysis (ROPA) with the top five production (positive) and loss (negative) reactions in the model for OH (top), HO_2_ (middle), and HO_*x*_ (bottom).

In comparison to OH, there are a variety of HO_2_ sources during the day. The sum of HCHO reactions contributes the most to HO_2_ production when there is available sunlight, although OH + CO and CH_3_O + O_2_ are also important formation mechanisms. Formaldehyde comes from a mixture of sources in Fairbanks, including local wood burning, heating oil combustion, traffic emissions, and secondary formation during aging of polluted air masses.^53^ The loss of HO_2_ is dominated by the reaction with NO to form OH. The formation of PNA is the second-largest loss term for HO_2_. At night, HO_2_ formation from PNA is greater than its loss, meaning that there is a net production of HO_2_ from PNA. A similar behavior of PNA was reported during the COBRA campaign in rural Canada.^17^ The net production of HO_2_ via PNA then contributes to OH production at night through the reaction with NO. However, if the residence time of PNA is short, then, this nocturnal production pathway breaks down.

When the lifetime of PNA with respect to physical loss was set to approximately 1 h in our sensitivity analysis, there was only minor impact on midday HO_*x*_ concentrations, but the impact on nighttime HO_2_ concentration and the budget was large (Figure 6). With the PNA residence time reduced, the nocturnal chemistry is suppressed and HO_2_ concentration goes to zero. The OH budget is also impacted, as there is no OH produced from HO_2_ at night, but the OH concentrations are similar between the two simulations. The physical residence time is largely determined by horizontal advection and mixing with air above the polluted surface layer. Residence time is expected to vary between stable, polluted periods and less stable, cleaner periods. While the sustained production of HO_2_ via PNA was also seen in rural Canada in the winter,^17^ the lack of PNA or HO*_*x*_* measurements to constrain our model makes it difficult to verify how important PNA cycling is in Fairbanks. Adjusting the *k*_phys_ of PNA implies that the physical residence time of PNA is the key determinant of its role in the nighttime HO_*x*_ budget.

**Figure 6:**
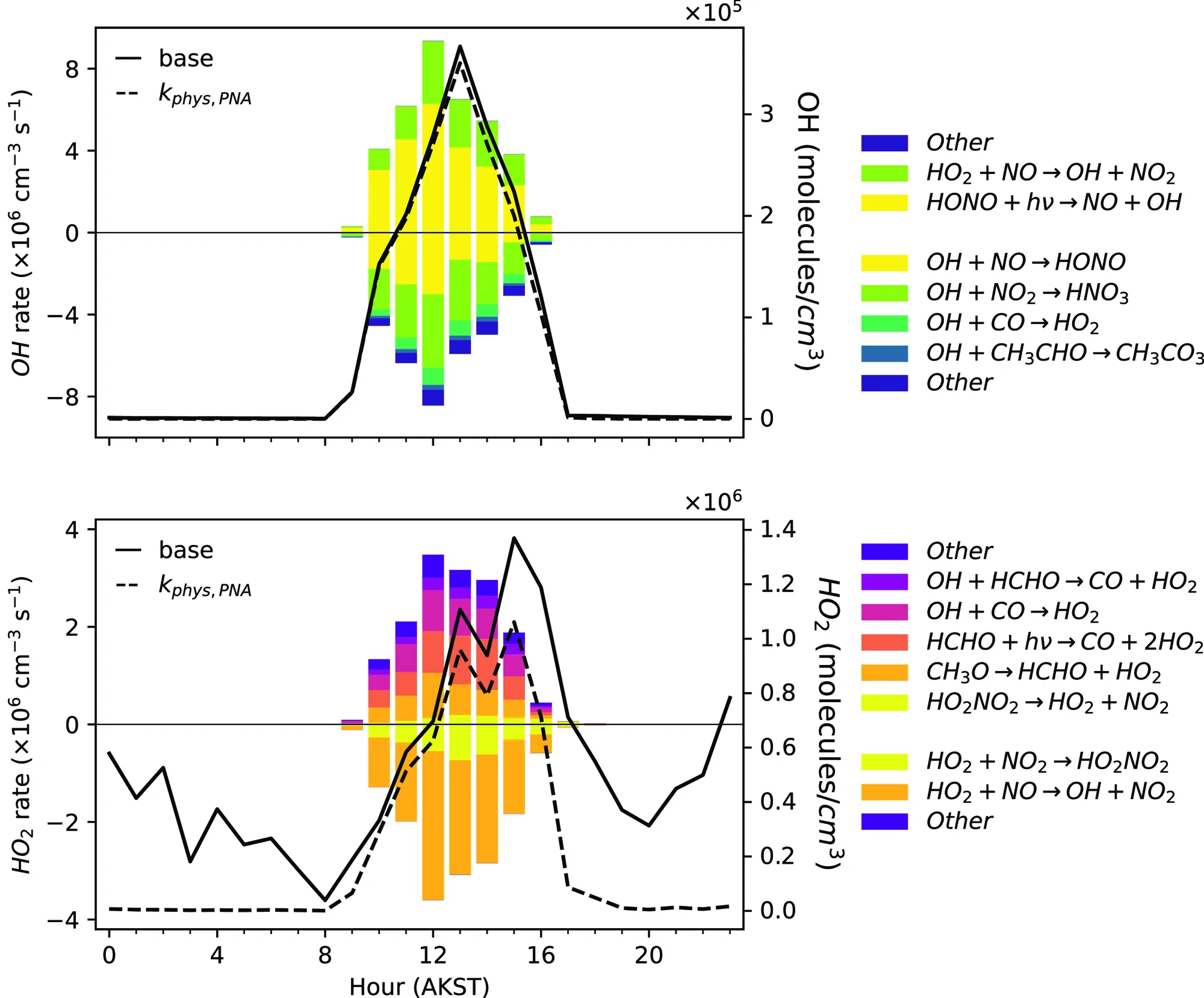
ROPAs (left *y*-axis) of the largest production (positive) and loss (negative) reactions in the chemical mechanism for OH (top) and HO_2_ (bottom) and concentration (right *y*-axis) of OH and HO_2_ for the base simulation (solid line) and the simulation with *k*_phys,PNA_ set to a residence time of approximately 1 h (dashed line).

The third row of Figure 5 shows the net total HO*_*x*_* budget. The reactions that recycle between OH and HO_2_, such as HO_2_ + NO → OH + NO_2_, do not contribute to net HO_*x*_ loss or production. HONO photolysis is the dominant net source of HO_*x*_, contributing on average 64% to daytime HO_*x*_ production. Photolysis of HONO is an important HO*_*x*_* source in other winter locations, for example, Beijing,^10,11^ Boulder, CO,^15^ and New York City.^14^ In our model, chemical HONO production occurs through two reactions: NO + OH and the heterogeneous reaction of NO_2_ with particles. The contribution of the NO_2_ heterogeneous reaction to HONO concentration is small in our simulations, although this may be due to the simplification of the reaction with a constant uptake coefficient. The reaction between NO and OH contributes more to HONO but is often less than the amount of HONO lost via photolysis, as demonstrated in Figure 5. This implies that other processes in addition to chemical production are important for controlling the abundance of HONO in Fairbanks, such as direct local emissions, transport of existing HONO from regional sources, or additional heterogeneous production processes.

Alkene ozonolysis was reported in previous campaigns such as PUMA and BEST-ONE as being an important HO*_*x*_* production pathway,^11,89^ but this pathway was not significant in our model. The dominant sink of HO*_*x*_* in our model is OH + NO_2_ → HNO_3_, accounting for 33% of total HO*_*x*_* loss on average and up to 50% at midday. Formation of HNO_3_ was the largest sink of HO*_*x*_* during the BEST-ONE campaign, near Beijing^11^ and in New York City during the winter PMTACS-NY campaign.^14^ Overall, our modeled HO*_*x*_* budget is dominated by features from the OH budget, while HO_2_ formation and loss pathways are small contributions.

#### 3.2.1 Sensitivity of the Model Budget to Constrained Inputs

Here, we present results of the sensitivity experiments to examine the impacts of key assumptions made in our box modeling framework (Table 2).

**Table 2:** Sensitivity of Midday HO_*x*_ Concentration to Model Perturbations

base simulation input	modification	ΔOH (%)	ΔHO_2_ (%)
*k*_phys_ = 1.16 × 10^–5^s^–1^ (τ_phys_ ≈ 1 day)	*k*_phys_ = 2.78 × 10^–4^ *s* ^–1^	–3.00	–10.70
	(τ_phys_ ≈ 1hr)		
	*k*_phys_ = 2.31 × 10^–6^ *s* ^–1^	1.39	5.82
	(τ_phys_ ≈ 5d*ay*)		
	*k*_phys,PNA_ = 2.78 × 10^–4^ *s* ^–1^	–3.12	–7.19
	(τ_phys,PNA_ ≈ 1hr)		
[HONO] from DOAS	1.2 × [HONO]	14.02	7.87
	0.8 × [HONO]	–14.10	–7.94
RH = 72%	RH = 30%	–0.07	–0.03
	RH = 60%	–0.02	–0.01
	RH = 90%	0.03	0.01
γ_OH_ = 1.0	γ_OH_ = 0.0	0.50	0.30
γ_HO_2__ = 0.1	γ_HO_2__ = 0.01	0.11	0.24
	γ_HO_2__ = 0.001	0.12	0.26
PTR-ToF-MS-identified VOCs	isoprene	–0.14	9.22
	*C*_2_–*C*_4_ alkanes/alkenes	–11.19	58.21

One uncertainty to our analysis of the HO_*x*_ budget during ALPACA is the limited available VOC measurements. When we constrain with additional estimated *C*_2_–*C*_4_ alkanes and alkenes, midday OH concentration decreases (−11% from the base model) and midday HO_2_ concentration increases (+58%). The decrease in OH concentration is due to additional reactivity with propene and ethene, which become the third and fourth most important OH loss mechanisms, respectively (Figure 7). The addition of the *C*_2_–*C*_4_ alkanes and alkenes also increased OH production from HO_2_+NO, as HO_2_ concentration is increased, but no other OH production reactions were significantly affected. In comparison, the addition of the *C*_2_–*C*_4_ alkanes and alkenes enhanced HO_2_ production during the day but did not contribute to additional loss except through the HO_2_+NO reaction. The decompositions of the 1-hydroxypropane-2-yloxy radical and the 2-hydroxyethoxy radical, formed from the oxidation of propene and ethene by OH, are the third and fourth largest contributors to HO_2_ production with the additional VOCs, respectively.

**Figure 7:**
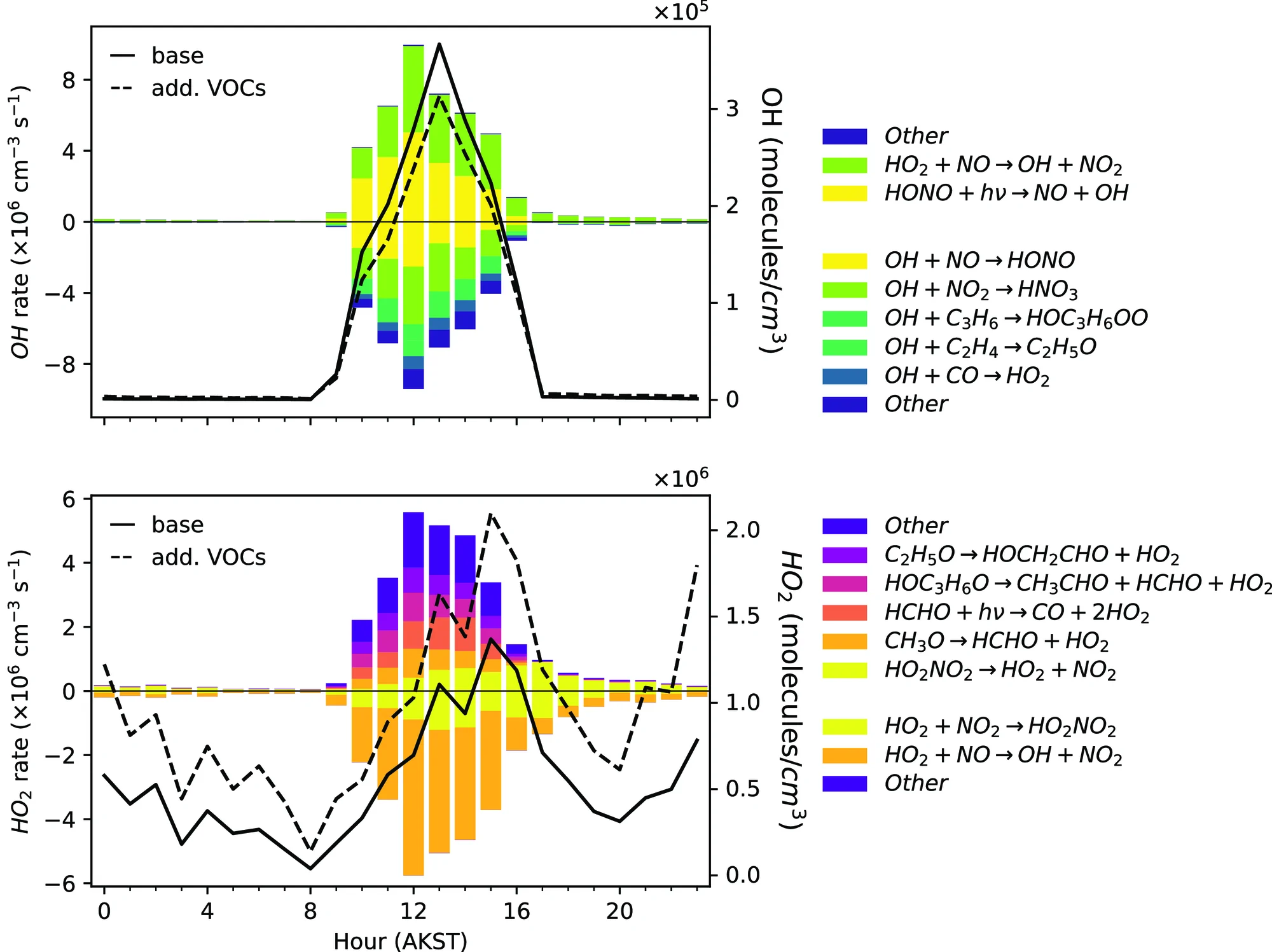
ROPAs (left *y*-axis) of the largest production (positive) and loss (negative) reactions in the chemical mechanism for OH (top) and HO_2_ (bottom) and concentration (right *y*-axis) of OH and HO_2_ for the base simulation (solid line) and the simulation with the updated VOC mechanism and constrained to additional VOC concentrations (add. VOC) (dashed line).

Previous studies using HO_*x*_ box modeling have argued that missing VOC measurements may help explain model-observation discrepancies, for example, by explaining missing OH reactivity.^21,26^ However, some analyses in polluted winter conditions have found that short-chain alkane and alkene species are a small contribution to OH reactivity.^10,21^ Our sensitivity analysis suggests that ethene and propene contribute to OH loss in Fairbanks, but short-chain alkanes do not. Missing alkene ozonolysis has also been proposed to explain OH underestimates compared to observations,^22,89^ but we find that OH production is not significantly impacted by additional reactions. This is likely due to the low ozone concentrations during the ALPACA period. Overall, we find that our model is sensitive to available VOC measurements that are used to constrain the simulations, specifically, short-chain alkenes.

Adding isoprene to the model constraints had a mixed effect on HO_*x*_ concentrations at midday. OH changed by −0.14% on average compared to the base simulation and HO_2_ increased by 9.2% (Table 2). This increase is due to additional HO_2_ production reactions: there are 464 reactions that contribute to the ‘Other’ category for HO_2_ production in the IC simulation compared to 216 in the base simulation. The largest contributor to the ‘Other’ category in the IC simulation is the decomposition of the isoprene derivative ISOPBO (1-methyl-1-vinyl-2-hydroxyethoxy radical, CH_2_(OH)­CO­(CH_3_)­CHCH_2_). This reaction did not contribute to HO_2_ production in the base simulation. These additional HO_2_ production pathways increase total HO_2_ concentration, which then increases OH concentration by reaction with NO. The increased production of OH is offset by the additional OH lost through reaction with isoprene and its derivatives, resulting in the small, negative percent difference in midday concentration.

The adjustments to physical loss rate had a small mean impact on the midday HO_*x*_ concentrations. As expected, a faster *k*_phys_ reduced HO_*x*_ concentrations and a slower *k*_phys_ increased HO_*x*_ concentrations. During the day, when production of HO_*x*_ is high, changes in *k*_phys_ have a small relative impact on ambient concentrations, and hourly mean percent difference for both OH and HO_2_ concentrations is small. The impact on HO_2_ is greater than that on OH. This is in part because there are more species that contribute to HO_2_ production than OH production, which is driven by constrained HONO concentration. A change in *k*_phys_ affects the concentrations of HO_2_ reactants such as PNA, CO, and many of the species in the “Other” category, resulting in a large cumulative impact.

Model-simulated midday HO_*x*_ concentrations display high sensitivity to uncertainty in the input HONO concentration because of the importance of HONO in the OH budget. A 20% increase in HONO concentration (1.2 × [HONO]) results in an increase in midday OH, which is slightly sublinear (a 14.0% increase in OH concentration is produced by a 20% increase in HONO input). This behavior results from a 7.9% increase in HO_2_ from cycling between OH and HO_2_. A 20% decrease in HONO input (0.8 × [HONO]) also results in an 14.1% decrease in OH concentration and 7.9% decrease in HO_2_. There is uncertainty if the HONO concentration aloft is representative of the surface conditions during weakly stable periods. These sensitivity experiments highlight the need for further study of HONO in the Arctic with colocated measurements.

Of the parameters tested, relative humidity had the smallest impact on HO_*x*_ concentrations. Varying relative humidity from 72% in the base case to 30, 60, or 90% resulted in less than ±0.1% difference in total HO_*x*_ concentrations on average. This result emphasizes the minor role of [Disp-formula eq2] as a source of OH in Fairbanks winters (Figure 5). Because of the minimal importance of [Disp-formula eq2], there is little impact of using a fixed average RH value and calculating the water vapor mixing ratio from interpolated hourly pressure measurements on our model results.

The difference in HO_2_ concentration to changes in γ_*HO*_2__ was less than 3% between the smallest and largest uptake values, which implies that HO_2_ loss in Fairbanks during winter is not sensitive to particle uptake. The uptake reaction in the model is included in the ‘Other’ category, which in Figure 5 is a small contributor to total HO_2_ loss when γ_HO_2__ = 0.1. Previous modeling studies of springtime subarctic conditions have found that HO_2_ uptake to particles accounts for up to 30% of the HO_*x*_ loss.^65,90^ However, the lower NO_*x*_ concentrations in these studies compared to the NO_*x*_ concentration measured during ALPACA mean that other loss reactions such as uptake become more competitive. In addition, γ_HO_2__ depends on several factors^65,67,68^ and uptake values representative of the spring or summer may not be ideal for Arctic winter. Concentrations of OH and HO_2_ in our analysis were also not sensitive to γ_OH_. OH concentrations changed by less than 1% on average between simulations with γ_OH_ = 1 and γ_OH_ = 0, indicating that OH in our model is not sensitive to reactive uptake.

#### 3.2.2 Budget in the Clean and Polluted Periods

While Figure 5 shows the diel cycle of the major chemical reactions contributing to HO_*x*_ production and loss in Fairbanks, Figures 8 and 9 demonstrate that there are large differences in the OH and HO_2_ budgets between the clean and polluted periods. There is enhanced OH production in the polluted period relative to the clean period and the campaign average in Figure 5. This increase is primarily from enhanced HONO photolysis despite the low-light conditions. There is also an increase in recycling from HO_2_ via a reaction with excess NO. A few other reactions have appreciable contributions to OH production during either period. There is a slight increase in the level of OH production at night in the clean period relative to that in the polluted period. The OH production to loss ratio (P/L) for Period 1 is 1.01, meaning that the enhanced production during the polluted event is balanced by an enhanced loss. Recycling of OH back into HONO is the dominant loss mechanism in the polluted period, followed by the formation of HNO_3_ by reaction with NO_2_. During Period 2, there is excess production relative to loss of OH (P/L = 1.22). The formation of HNO_3_ is the dominant loss mechanism, while the reaction with NO to reform HONO is minimal because there is less available NO during the clean period. In comparison, in the polluted period, NO_*x*_ emissions are trapped in the inversion, and there is minimal mixing with clean air from outside Fairbanks. Ozone is depleted through titration with NO to form NO_2_; excess NO is therefore available to react with OH among other species.

**Figure 8:**
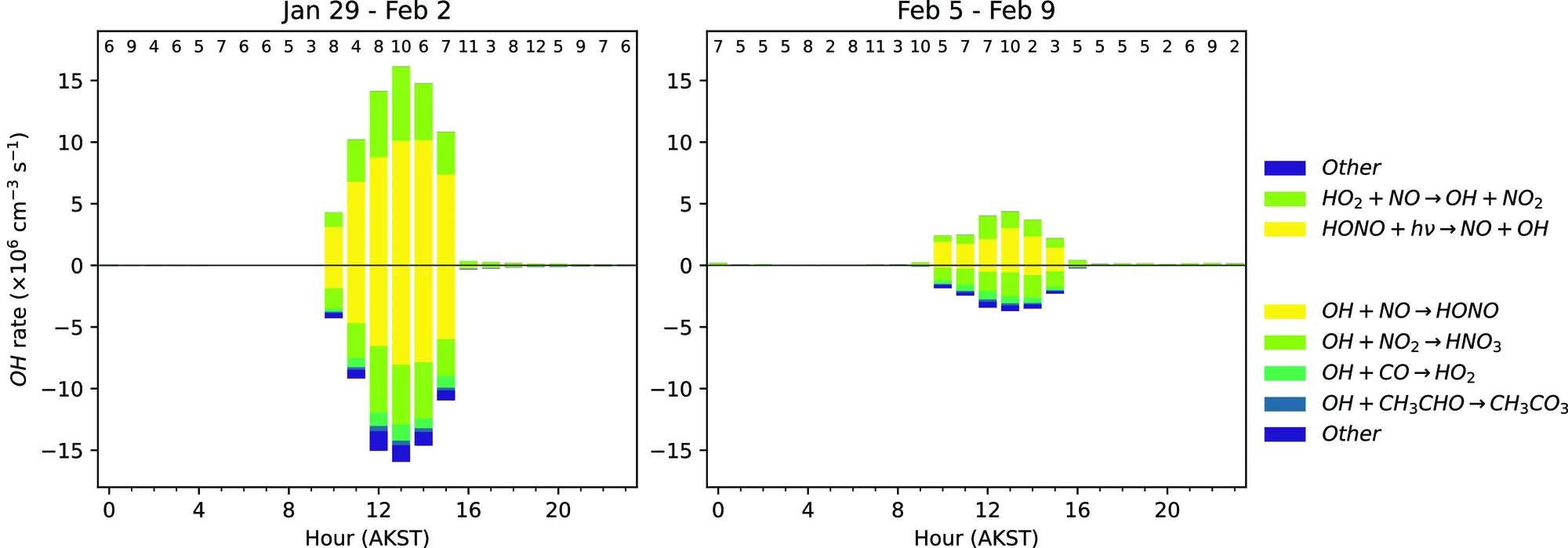
ROPAs of the largest production (positive) and loss (negative) reactions in the chemical mechanism for OH, comparing two time periods during the campaign. Jan 29 to Feb 02 is a polluted period during a strong surface-based inversion, while Feb 05 to Feb 09 was clean. We only explicitly show reactions that are significant contributors to either loss or production. All other reactions are included in ‘Other’. The number of samples in each hour bin is included along the top *y*-axis.

**Figure 9:**
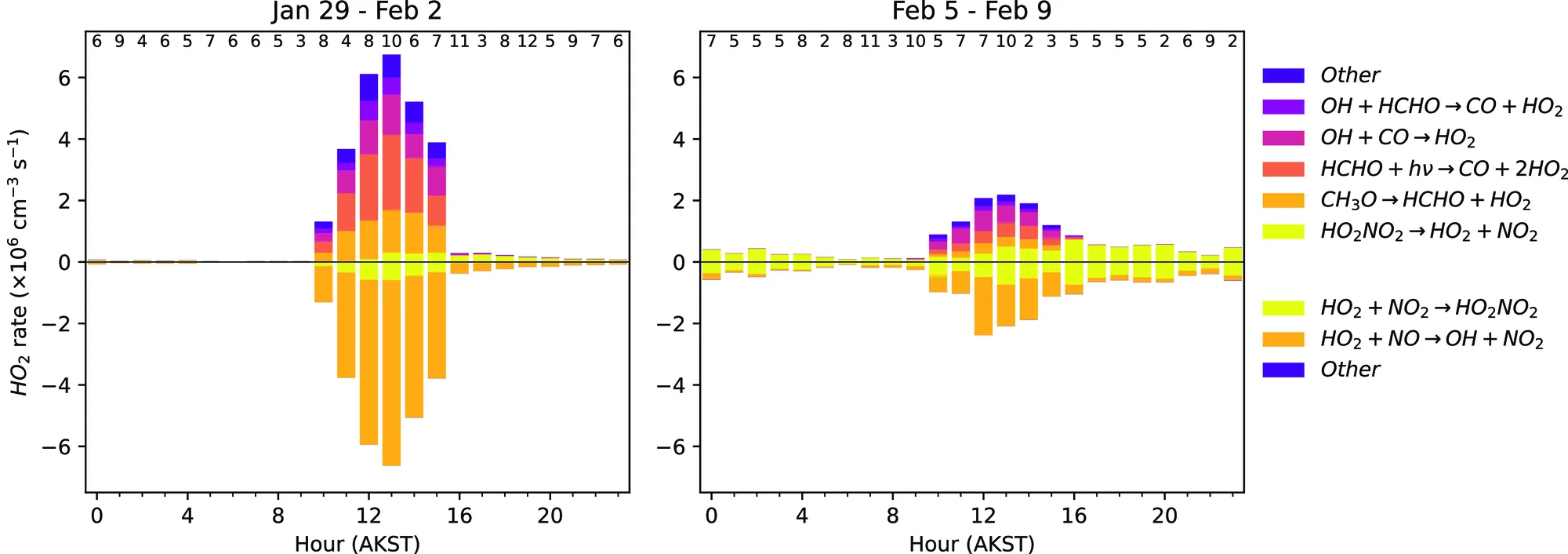
ROPAs of the largest production (positive) and loss (negative) reactions in the chemical mechanism for HO_2_, comparing two time periods during the campaign. Jan 29 to Feb 02 is a polluted period during a strong surface-based inversion, while Feb 05 to Feb 09 was clean. We only explicitly show reactions that are significant contributors to either loss or production. All other reactions are included in ‘Other’. The number of samples in each hour bin is included along the top *y*-axis.

There is enhanced daytime HO_2_ production in the polluted period relative to the clean period (Figure 9). However, at night, there is a distinct increase in HO_2_ production in the clean period compared to the polluted period. This production is driven by decomposition of PNA. The enhanced HO_2_ production at night does not, however, outweigh the enhanced loss during Period 2 (*P*/*L* = 0.91). The nocturnal production decreases throughout the evening, reaching a minimum shortly before sunrise. In the early morning, loss outweighs production in the clean period, resulting in the local minimum in HO_2_ concentration in Figure 4. In the polluted period, HO_2_ production and loss are balanced (*P*/*L* = 1.01). Cycling between PNA and HO_2_ + NO_2_ is not a large part of the budget during Period 1. Production of HO_2_ during the polluted period is dominated by production from HCHO, methoxy radical (CH_3_O), and smaller individual contributions from reactions lumped into ‘Other’. The lack of production via PNA in the polluted period at night results in near-zero HO_2_ concentration for much of the evening and early morning. The differences in PNA chemistry between the two periods may in part be driven by temperature differences. Although the PNA concentration is similar in the clean and polluted periods, during the clean period, the average temperature is warmer by 10 K on average. During the polluted period an average temperature of 245 K corresponds to a PNA lifetime against thermal decomposition of approximately 11 h. The PNA lifetime against thermal decomposition reduces to approximately 2 h at 255 K during the warmer, cleaner period. This difference contributes to enhanced thermal decomposition of PNA and a sustained HO_2_ concentration at night during the cleaner period.

## 4 Conclusions

We have explored the HO_*x*_ budget of a polluted wintertime Arctic urban environment and how it compares with previous midlatitude wintertime observations and modeling of HO_*x*_. Model-simulated HO_*x*_ concentrations in Fairbanks are lower on average than wintertime measurements made in other urban areas such as Beijing. Despite limited solar radiation at short wavelengths and the large solar zenith angle, the photolysis of HONO and formaldehyde occurring at longer wavelengths sustains the oxidizing capacity in Fairbanks. Chemical production alone cannot explain HONO concentration in Fairbanks, and there is uncertainty in HONO emission sources and heterogeneous chemistry, which can make the modeling of OH in the Arctic challenging.^91–96^ Future work to better understand HONO production in the Arctic could therefore improve the model representation of OH concentrations.

The HO_*x*_ budget in our model was impacted by the meteorological conditions in Fairbanks, with strongly stable inversion events having a different atmospheric composition and chemistry from weakly stable periods. For our two case study periods, the strongly stable period was more polluted, and this period had lower HO_2_ and O_3_ concentrations compared to the weakly stable, clean period. There is large diel variability in the HO_2_ concentration throughout our model simulations. The peaks in HO_2_ concentration are at opposite times of day in the clean versus polluted periods driven by the different chemical and meteorological regimes. During the clean period, cycling between PNA (HO_2_NO_2_) and HO_2_ + NO_2_ is large and contributes to the build-up of HO_2_ concentration at night. This may be due in part to the warmer temperatures promoting the thermal decomposition of the PNA. However, this finding is extremely sensitive to assumptions regarding physical controls of the PNA atmospheric residence time. In comparison, nocturnal chemistry is minimal during the polluted period, and there is no accumulation of HO_2_ at night. HO_2_ production during the day is driven by formaldehyde photolysis and oxidation, especially during the polluted periods. Despite the differences in the HO_2_ budget and higher OH reactivity during the polluted period, the OH concentration in the two periods is similar. HONO, and to some extent formaldehyde through HO_2_, sustain OH concentration in both periods, and lower NO_*x*_ concentrations in the clean period limit OH loss. The largest chemical sink of OH on average is the reaction with NO_2_ to form HNO_3_, which may be considered a terminal loss of NO_*x*_. However, during the heavily polluted surface-based inversion event from January 29 to February 2, recycling of OH and NO into HONO was the greatest sink of OH. This reaction is not a permanent sink of OH; thus, this reaction may help sustain OH concentrations during pollution events.

When including a measured C_5_H_9_^+^ fragment as isoprene in the model and adding estimated concentrations of short-chain alkanes and alkenes as constraints, there were significant changes in the HO_*x*_ budget. Adding isoprene and alkenes increased loss of OH through reactions with these species but increased HO_2_ via multiple production reactions, including decomposition of peroxy radicals produced by OH oxidation. This indicates that DSMACC is sensitive to included VOCs and reveals that the HO_*x*_ oxidation of organics is relevant in Arctic urban locations. As there were no measurements of OH concentration or reactivity made during the ALPACA campaign, we cannot directly compare our model results, and our modeling of OH reactivity is limited by measured VOC species. Previous model-observation comparisons of the HO_*x*_ budget have reported an underestimate of OH reactivity that may be due to missing VOC measurements.^11,14,21,26^

Our additional sensitivity analyses demonstrated that model-simulated HO_*x*_ is sensitive to the physical loss rate and constrained HONO concentration but not sensitive to relative humidity or uptake. A source of uncertainty comes from our representation of physical loss mechanisms such as mixing. We use a lumped rate to represent the loss through deposition and transport. The midday HO_*x*_ concentrations were moderately impacted by modification of the *k*_*phys*_ parameter, but nocturnal chemistry was greatly affected, especially PNA cycling. These results reveal the uncertainty in our model with respect to physical loss and for species that we do not constrain with measured concentrations. Future field studies in Arctic polluted environments that include colocated measurements of PNA, HONO, VOCs, and radical species are needed to corroborate our modeling results.

## Supplementary Material



## Notes

Comparison between normalized kernel density estimate (KDE) distributions of whole-campaign ALPACA CO measurements and subset of CO measurements used in DSMACC modeling (PDF)

## ⋈

Department of Earth and Environmental Sciences, The University of Manchester, Manchester M13 9PL, U.K

## ⧓

Center for Atmospheric and Environmental Chemistry, Aerodyne Research Inc., Billerica, Massachusetts 01821, USA

## References

[1] Atkinson R. (2000). Atmospheric
chemistry of VOCs and NOx. Atmos. Environ..

[2] Levy H. (1971). Normal Atmosphere:
Large Radical and Formaldehyde Concentrations Predicted. Science.

[3] Atkinson R., Arey J. (2003). Atmospheric Degradation of Volatile
Organic Compounds. Chem. Rev..

[4] Ziemann P. J., Atkinson R. (2012). Kinetics, products,
and mechanisms of secondary organic
aerosol formation. Chem. Soc. Rev..

[5] Rigby M., Montzka S. A., Prinn R. G. (2017). Role of atmospheric
oxidation in recent methane growth. Proc. Natl.
Acad. Sci. U. S. A..

[6] Zhao Y., Saunois M., Bousquet P. (2020). On the role of trend
and variability in the hydroxyl radical (OH) in the global methane
budget. Atmos. Chem. Phys..

[7] Spivakovsky C. M., Logan J. A., Montzka S. A., Balkanski Y. J., Foreman-Fowler M., Jones D. B. A., Horowitz L. W., Fusco A. C., Brenninkmeijer C. A.
M., Prather M. J., Wofsy S. C., McElroy M. B. (2000). Three-dimensional
climatological distribution of tropospheric OH: Update and evaluation. J. Geophys. Res.:Atmos..

[8] Lelieveld J., Gromov S., Pozzer A., Taraborrelli D. (2016). Global tropospheric
hydroxyl distribution, budget and reactivity. Atmos. Chem. Phys..

[9] Beyersdorf A. J., Blake D. R., Swanson A., Meinardi S., Rowland F. S., Davis D. (2010). Abundances and variability of tropospheric
volatile organic compounds
at the South Pole and other Antarctic locations. Atmos. Environ..

[10] Slater E. J., Whalley L. K., Woodward-Massey R. (2020). Elevated levels of OH
observed in haze events during wintertime in central Beijing. Atmos. Chem. Phys..

[11] Tan Z., Rohrer F., Lu K. (2018). Wintertime photochemistry
in Beijing: observations of RO_
*x*
_ radical
concentrations in the North China Plain during the BEST-ONE campaign. Atmos. Chem. Phys..

[12] Stone D., Whalley L. K., Heard D. E. (2012). Tropospheric OH
and HO2 radicals:
field measurements and model comparisons. Chem.
Soc. Rev..

[13] Ehhalt D.
H., Rohrer F. (2000). Dependence
of the OH concentration on solar UV. J. Geophys.
Res.:Atmos..

[14] Ren X., Brune W. H., Mao J. (2006). Behavior of OH and HO2
in the winter atmosphere in New York City. Atmos.
Environ..

[15] Kim S., VandenBoer T. C., Young C. J. (2014). The primary and recycling
sources of OH during the NACHTT-2011 campaign: HONO as an important
OH primary source in the wintertime. J. Geophys.
Res.:Atmos..

[16] Ma X., Tan Z., Lu K., Yang X., Liu Y., Li S., Li X., Chen S., Novelli A., Cho C., Zeng L., Wahner A., Zhang Y. (2019). Winter photochemistry in Beijing:
Observation and model simulation of OH and HO2 radicals at an urban
site. Sci. Total Environ..

[17] Edwards P., Evans M. J., Commane R., Ingham T., Stone D., Mahajan A. S., Oetjen H., Dorsey J. R., Hopkins J. R., Lee J. D., Moller S. J., Leigh R., Plane J. M. C., Carpenter L. J., Heard D. E. (2011). Hydrogen oxide photochemistry in
the northern Canadian spring time boundary layer. J. Geophys. Res.:Atmos..

[18] Van
Overmeiren P., Demeestere K., Mangold A., Delcloo A., Van Langenhove H., Walgraeve C. (2023). Year-round measurement of atmospheric
volatile organic compounds using sequential sampling in Dronning Maud
Land, East-Antarctica. Atmos. Environ..

[19] Cai C., Hogrefe C., Katsafados P., Kallos G., Beauharnois M., Schwab J. J., Ren X., Brune W. H., Zhou X., He Y., Demerjian K. L. (2008). Performance
evaluation of an air quality forecast modeling
system for a summer and winter season - Photochemical oxidants and
their precursors. Atmos. Environ..

[20] Kanaya Y., Cao R., Kato S., Miyakawa Y., Kajii Y., Tanimoto H., Yokouchi Y., Mochida M., Kawamura K., Akimoto H. (2007). Chemistry
of OH and HO_2_ radicals observed at Rishiri Island, Japan,
in September 2003: Missing daytime sink of HO_2_ and positive
nighttime correlations with monoterpenes. J.
Geophys. Res.:Atmos..

[21] Yoshino A., Sadanaga Y., Watanabe K., Kato S., Miyakawa Y., Matsumoto J., Kajii Y. (2006). Measurement of total OH reactivity
by laser-induced pump and probe techniquecomprehensive observations
in the urban atmosphere of Tokyo. Atmos. Environ..

[22] Cho C., Fuchs H., Hofzumahaus A. (2023). Experimental chemical
budgets of OH, HO_2_, and RO_2_ radicals in rural
air in western Germany during the JULIAC campaign 2019. Atmos. Chem. Phys..

[23] Mauldin R. L., Cantrell C. A., Zondlo M. A., Kosciuch E., Ridley B. A., Weber R., Eisele F. E. (2003). Measurements of
OH, H_2_ SO_4_, and MSA during Tropospheric Ozone
Production About
the Spring Equinox (TOPSE). J. Geophys. Res.:Atmos..

[24] Brune W. H., Miller D. O., Thames A. B. (2020). Exploring
Oxidation
in the Remote Free Troposphere: Insights From Atmospheric Tomography
(ATom). J. Geophys. Res.:Atmos..

[25] Miller D. O., Brune W. H. (2022). Investigating the Understanding of
Oxidation Chemistry
Using 20 Years of Airborne OH and HO_2_ Observations. J. Geophys. Res.:Atmos..

[26] Dolgorouky C., Gros V., Sarda-Esteve R., Sinha V., Williams J., Marchand N., Sauvage S., Poulain L., Sciare J., Bonsang B. (2012). Total OH reactivity measurements in Paris during the
2010 MEGAPOLI winter campaign. Atmos. Chem.
Phys..

[27] Heard D. E., Carpenter L. J., Creasey D. J., Hopkins J. R., Lee J. D., Lewis A. C., Pilling M. J., Seakins P. W., Carslaw N., Emmerson K. M. (2004). High levels
of the hydroxyl radical in the winter urban
troposphere. Geophys. Res. Lett..

[28] Sinha V., Williams J., Diesch J. M., Drewnick F., Martinez M., Harder H., Regelin E., Kubistin D., Bozem H., Hosaynali-Beygi Z., Fischer H., Andrés-Hernández M. D., Kartal D., Adame J. A., Lelieveld J. (2012). Constraints
on instantaneous ozone production rates and regimes during DOMINO
derived using in-situ OH reactivity measurements. Atmos. Chem. Phys..

[29] Rohrer F., Berresheim H. (2006). Strong correlation between levels
of tropospheric hydroxyl
radicals and solar ultraviolet radiation. Nature.

[30] Birmili W., Berresheim H., Plass-Dülmer C., Elste T., Gilge S., Wiedensohler A., Uhrner U. (2003). The Hohenpeissenberg aerosol formation
experiment (HAFEX): a long-term study including size-resolved aerosol,
H_2_SO_4_, OH, and monoterpenes measurements. Atmos. Chem. Phys..

[31] Walker H. M., Stone D., Ingham T. (2015). Night-time measurements
of HO_
*x*
_ during the RONOCO project and analysis
of the sources of HO_2_. Atmos. Chem.
Phys..

[32] Cho C., Hofzumahaus A., Fuchs H., Dorn H.-P., Glowania M., Holland F., Rohrer F., Vardhan V., Kiendler-Scharr A., Wahner A., Novelli A. (2021). Characterization of a chemical modulation
reactor (CMR) for the measurement of atmospheric concentrations of
hydroxyl radicals with a laser-induced fluorescence instrument. Atmos. Meas. Tech..

[33] Kanaya Y., Cao R., Akimoto H., Fukuda M., Komazaki Y., Yokouchi Y., Koike M., Tanimoto H., Takegawa N., Kondo Y. (2007). Urban photochemistry
in central Tokyo: 1. Observed and modeled OH and HO_2_ radical
concentrations during the winter and summer of 2004. J. Geophys. Res.:Atmos..

[34] Yang Y., Wang Y., Huang W., Yao D., Zhao S., Wang Y., Ji D., Zhang R., Wang Y. (2021). Parameterized
atmospheric oxidation capacity and speciated OH reactivity over a
suburban site in the North China Plain: A comparative study between
summer and winter. Sci. Total Environ..

[35] Simpson W. R., Mao J., Fochesatto G. J. (2024). Overview of the Alaskan
Layered Pollution and Chemical Analysis (ALPACA) Field Experiment. ACS ES&T Air.

[36] ADEC . Air Quality Control Program Implementation Status: Fairbanks North Star Borough PM2.5 Nonattainment Area; Annual 2. 2022.

[37] Tran H. N., Mölders N. (2011). Investigations on meteorological conditions for elevated
PM2.5 in Fairbanks, Alaska. Atmos. Res..

[38] Wang Y., Hopke P. K. (2014). Is Alaska Truly
the Great Escape from Air Pollution?
- Long Term Source Apportionment of Fine Particulate Matter in Fairbanks,
Alaska. Aerosol Air Qual. Res..

[39] Farrell S. L., Pye H. O. T., Gilliam R., Pouliot G., Huff D., Sarwar G., Vizuete W., Briggs N., Duan F., Ma T., Zhang S., Fahey K. (2025). Predicted impacts of heterogeneous
chemical pathways on particulate sulfur over Fairbanks (Alaska), the
Northern Hemisphere, and the Contiguous United States. Atmos. Chem. Phys..

[40] Emmerson K. M., Evans M. J. (2009). Comparison of tropospheric gas-phase
chemistry schemes
for use within global models. Atmos. Chem. Phys..

[41] Stone D. (2010). HO_
*x*
_ observations over West Africa during
AMMA: impact of isoprene and NO_
*x*
_. Atmos. Chem. Phys..

[42] Jenkin M. E., Saunders S. M., Pilling M. J. (1997). The tropospheric degradation of volatile
organic compounds: a protocol for mechanism development. Atmos. Environ..

[43] Saunders S. M., Jenkin M. E., Derwent R. G., Pilling M. J. (2003). Protocol
for the
development of the Master Chemical Mechanism, MCM v3 (Part A): tropospheric
degradation of non-aromatic volatile organic compounds. Atmos. Chem. Phys..

[44] Sandu A., Sander R. (2006). Technical note: Simulating chemical systems in Fortran90
and Matlab with the Kinetic PreProcessor KPP-2.1. Atmos. Chem. Phys..

[45] Madronich, S. ; Flocke, S. Environmental Photochemistry; Hutzinger, O. ; Boule, P. , Eds.; Springer: Berlin, Heidelberg, 1999; Vol. 2/2L, pp 1–26.

[46] Heinlein L. M. D., He J., Sunday M. O. (2025). Surprisingly robust
photochemistry in subarctic particles during winter: evidence from
photooxidants. Atmos. Chem. Phys..

[47] Müller M., Eichler P., D’Anna B., Tan W., Wisthaler A. (2017). Direct Sampling
and Analysis of Atmospheric Particulate Organic Matter by Proton-Transfer-Reaction
Mass Spectrometry. Anal. Chem..

[48] Ijaz A., Temime-Roussel B., Kammer J., Mao J., Simpson W., Law K. S., D’Anna B. (2025). *In situ* measurements
of gas-particle partitioning of organic compounds in Fairbanks. Faraday Discuss..

[49] St
Clair J. M., Swanson A. K., Bailey S. A., Wolfe G. M., Marrero J. E., Iraci L. T., Hagopian J. G., Hanisco T. F. (2017). A new non-resonant
laser-induced fluorescence instrument for the airborne in situ measurement
of formaldehyde. Atmos. Meas. Tech..

[50] Shutter J. D., Allen N. T., Hanisco T. F., Wolfe G. M., Clair J. M., Keutsch F. N. (2019). A new laser-based
and ultra-portable
gas sensor for indoor and outdoor formaldehyde (HCHO) monitoring. Atmos. Meas. Tech..

[51] Cesler-Maloney M., Simpson W. R., Miles T., Mao J., Law K. S., Roberts T. J. (2022). Differences in Ozone and Particulate Matter Between
Ground Level and 20 m Aloft are Frequent During Wintertime Surface-Based
Temperature Inversions in Fairbanks, Alaska. J. Geophys. Res.:Atmos..

[52] Platt, U. ; Stutz, J. Differential Optical Absorption Spectroscopy; Physics of Earth and Space Environments; Springer Berlin Heidelberg: Berlin, Heidelberg, 2008.

[53] Ketcherside D. T., Yokelson R. J., Selimovic V. (2025). Wintertime Abundance
and Sources of Key Trace Gas and Particle Species in Fairbanks, Alaska. J. Geophys. Res.:Atmos..

[54] Brett N., Law K. S., Arnold S. R. (2025). Investigating processes
influencing simulation of local Arctic wintertime anthropogenic pollution
in Fairbanks, Alaska, during ALPACA-2022. Atmos.
Chem. Phys..

[55] Lan, X. ; Mund, J. ; Crotwell, A. ; Thoning, K. ; Moglia, E. ; Madronich, M. ; Baugh, K. ; Petron, G. ; Crotwell, M. ; Neff, D. ; Wolter, S. ; Mefford, T. ; DeVogel, S. Atmospheric Methane Dry Air Mole Fractions from the NOAA GML Carbon Cycle Cooperative Global Air Sampling Network. 2025, pp 1983–2024.

[56] Petron, G. ; Crotwell, A. ; Crotwell, M. ; Kitzis, D. ; Madronich, M. ; Mefford, T. ; Moglia, E. ; Mund, J. ; Neff, D. ; Thoning, K. ; Wolter, S. Atmospheric Hydrogen Dry Air Mole Fractions from the NOAA GML Carbon Cycle Cooperative Global Air Sampling Network20092024 2025 https://www.esrl.noaa.gov/gmd/ccgg/arc/?id=132&doi=10.15138/wp0w-ez08.

[57] Li S., Song W., Zhan H. (2021). Contribution of Vehicle
Emission and NO _2_ Surface Conversion to Nitrous Acid (HONO)
in Urban Environments: Implications from Tests in a Tunnel. Environ. Sci. Technol..

[58] Davies J. F., Wilson K. R. (2015). Nanoscale interfacial gradients formed by the reactive
uptake of OH radicals onto viscous aerosol surfaces. Chem. Sci..

[59] Kessler S. H., Nah T., Daumit K. E., Smith J. D., Leone S. R., Kolb C. E., Worsnop D. R., Wilson K. R., Kroll J. H. (2012). OH-Initiated Heterogeneous
Aging of Highly Oxidized Organic Aerosol. J.
Phys. Chem. A.

[60] Li Z., Smith K. A., Cappa C. D. (2018). Influence of relative humidity on
the heterogeneous oxidation of secondary organic aerosol. Atmos. Chem. Phys..

[61] Li J., Forrester S. M., Knopf D. A. (2020). Heterogeneous oxidation of amorphous
organic aerosol surrogates by O_3_, NO_3_, and OH
at typical tropospheric temperatures. Atmos.
Chem. Phys..

[62] George I. J., Slowik J., Abbatt J. P. D. (2008). Chemical aging of ambient organic
aerosol from heterogeneous reaction with hydroxyl radicals. Geophys. Res. Lett..

[63] Kroll J. H., Lim C. Y., Kessler S. H., Wilson K. R. (2015). Heterogeneous Oxidation
of Atmospheric Organic Aerosol: Kinetics of Changes to the Amount
and Oxidation State of Particle-Phase Organic Carbon. J. Phys. Chem. A.

[64] Waring C., King K. L., Bagot P. A. J., Costen M. L., McKendrick K. G. (2011). Collision
dynamics and reactive uptake of OH radicals at liquid surfaces of
atmospheric interest. Phys. Chem. Chem. Phys..

[65] Mao J., Jacob D. J., Evans M. J. (2010). Chemistry of hydrogen
oxide radicals (HO_
*x*
_) in the Arctic troposphere
in spring. Atmos. Chem. Phys..

[66] Thornton J. A., Jaeglé L., McNeill V. F. (2008). Assessing known pathways for HO_2_ loss in
aqueous atmospheric aerosols: Regional and global
impacts on tropospheric oxidants. J. Geophys.
Res.:Atmos..

[67] Matthews, P. S. J. Heterogeneous uptake of HO2 radicals onto atmospheric aerosols, Ph.D. Thesis; University of Leeds: Leeds, UK, 2014.

[68] Lakey P. S. J., Berkemeier T., Baeza-Romero M. T., Pöschl U., Shiraiwa M., Heard D. E. (2024). Towards a better
understanding of
the HO2 uptake coefficient to aerosol particles measured during laboratory
experiments. Environ. Sci.:Atmos..

[69] Jaeglé L., Jacob D. J., Brune W. H., Faloona I., Tan D., Heikes B. G., Kondo Y., Sachse G. W., Anderson B., Gregory G. L., Singh H. B., Pueschel R., Ferry G., Blake D. R., Shetter R. E. (2000). Photochemistry
of HO_
*x*
_ in the upper troposphere at northern
midlatitudes. J. Geophys. Res.:Atmos..

[70] Jacob D. (2000). Heterogeneous
chemistry and tropospheric ozone. Atmos. Environ..

[71] Zhou J., Murano K., Kohno N., Sakamoto Y., Kajii Y. (2020). Real-time
quantification of the total HO2 reactivity of ambient air and HO2
uptake kinetics onto ambient aerosols in Kyoto (Japan). Atmos. Environ..

[72] Taketani F., Kanaya Y., Pochanart P., Liu Y., Li J., Okuzawa K., Kawamura K., Wang Z., Akimoto H. (2012). Measurement
of overall uptake coefficients for HO_2_ radicals by aerosol
particles sampled from ambient air at Mts. Tai and Mang (China). Atmos. Chem. Phys..

[73] Kohno N., Zhou J., Li J., Takemura M., Ono N., Sadanaga Y., Nakashima Y., Sato K., Kato S., Sakamoto Y., Kajii Y. (2022). Impacts of missing OH reactivity
and aerosol uptake of HO2 radicals on tropospheric O3 production during
the AQUAS-Kyoto summer campaign in 2018. Atmos.
Environ..

[74] Dyson J. E., Whalley L. K., Slater E. J. (2023). Impact of HO_2_ aerosol uptake on radical levels and O_3_ production during
summertime in Beijing. Atmos. Chem. Phys..

[75] Coggon M. M., Stockwell C. E., Claflin M. S. (2024). Identifying and correcting
interferences to PTR-ToF-MS measurements of isoprene and other urban
volatile organic compounds. Atmos. Meas. Tech..

[76] Ernle L., Wang N., Bekö G., Morrison G., Wargocki P., Weschler C. J., Williams J. (2023). Assessment
of aldehyde contributions
to PTR-MS m/z 69.07 in indoor air measurements. Environ. Sci.: Atmos..

[77] Burkholder, J. B. ; Sander, S. P. ; Abbatt, J. ; Barker, J. R. ; Cappa, C. ; Crounse, J. D. ; Dibble, T. S. ; Huie, R. E. ; Kolb, C. E. ; Kurylo, M. J. ; Orkin, V. L. ; Percival, C. J. ; Wilmouth, D. M. ; Wine, P. H. JPL Publication 19–5; Jet Propulsion Laboratory: Pasadena, CA, 2019.

[78] Permar W., Wang Q., Selimovic V. (2021). Emissions of Trace Organic
Gases From Western U.S. Wildfires Based on WE-CAN Aircraft Measurements. J. Geophys. Res.:Atmos..

[79] Gordon T. D., Tkacik D. S., Presto A. A., Zhang M., Jathar S. H., Nguyen N. T., Massetti J., Truong T., Cicero-Fernandez P., Maddox C., Rieger P., Chattopadhyay S., Maldonado H., Maricq M. M., Robinson A. L. (2013). Primary Gas- and
Particle-Phase Emissions and Secondary Organic Aerosol Production
from Gasoline and Diesel Off-Road Engines. Environ.
Sci. Technol..

[80] Niu Z., Kong S., Zheng H., Hu Y., Zheng S., Cheng Y., Yao L., Liu W., Ding F., Liu X., Qi S. (2023). Differences in compositions
and effects of VOCs from
vehicle emission detected using various methods. Environ. Pollut..

[81] Wang M., Li S., Zhu R., Zhang R., Zu L., Wang Y., Bao X. (2020). On-road tailpipe emission characteristics
and ozone formation potentials
of VOCs from gasoline, diesel and liquefied petroleum gas fueled vehicles. Atmos. Environ..

[82] Shen X., Ni H., Lv T., Yao Z., Che H., Hao X., Wu B., Cao X., Li X., Zhou Q. (2025). Progress in the study
of the emission characteristics of intermediate and semivolatile organic
compounds from motor vehicles. J. Environ. Sci..

[83] Frey H. C. (2018). Trends
in onroad transportation energy and emissions. J. Air Waste Manage. Assoc..

[84] McDonald B. C., Gentner D. R., Goldstein A. H., Harley R. A. (2013). Long-Term Trends
in Motor Vehicle Emissions in U.S. Urban Areas. Environ. Sci. Technol..

[85] Pang Y., Fuentes M., Rieger P. (2015). Trends in selected ambient volatile
organic compound (VOC) concentrations and a comparison to mobile source
emission trends in California’s South Coast Air Basin. Atmos. Environ..

[86] Huo S., Zhang X., Xu W., Dang J., Xu F., Xie W., Tao C., Han Y., Liu X., Teng Z., Xie R., Cao X., Zhang Q. (2024). Updating vehicle VOCs emissions characteristics
under clean air actions in a tropical city of China. Sci. Total Environ..

[87] Gentner D. R., Isaacman G., Worton D. R., Chan A. W. H., Dallmann T. R., Davis L., Liu S., Day D. A., Russell L. M., Wilson K. R., Weber R., Guha A., Harley R. A., Goldstein A. H. (2012). Elucidating
secondary organic aerosol from diesel and
gasoline vehicles through detailed characterization of organic carbon
emissions. Proc. Natl. Acad. Sci..

[88] Hays M. D., Beck L., Barfield P., Lavrich R. J., Dong Y., Vander Wal R. L. (2008). Physical
and Chemical Characterization of Residential
Oil Boiler Emissions. Environ. Sci. Technol..

[89] Emmerson K. M., Carslaw N., Pilling M. J. (2005). Urban Atmospheric
Chemistry During
the PUMA Campaign 2: Radical Budgets for OH, HO2 and RO2. J. Atmos. Chem..

[90] Bloss W. J., Lee J. D., Johnson G. P., Sommariva R., Heard D. E., Saiz-Lopez A., Plane J. M. C., McFiggans G., Coe H., Flynn M., Williams P., Rickard A. R., Fleming Z. L. (2005). Impact
of halogen monoxide chemistry upon boundary layer OH and HO_2_ concentrations at a coastal site. Geophys.
Res. Lett..

[91] Zhang S., Sarwar G., Xing J., Chu B., Xue C., Sarav A., Ding D., Zheng H., Mu Y., Duan F., Ma T., He H. (2021). Improving the representation
of HONO chemistry in CMAQ and examining its impact on haze over China. Atmos. Chem. Phys..

[92] Wong K. W., Tsai C., Lefer B., Grossberg N., Stutz J. (2013). Modeling of daytime HONO vertical gradients during SHARP 2009. Atmos. Chem. Phys..

[93] Wang L., Chai J., Gaubert B., Huang Y. (2025). A review of
measurements
and model simulations of atmospheric nitrous acid. Atmos. Environ..

[94] Liu Y., Lu K., Li X., Dong H., Tan Z., Wang H., Zou Q., Wu Y., Zeng L., Hu M., Min K.-E., Kecorius S., Wiedensohler A., Zhang Y. (2019). A Comprehensive Model
Test of the HONO Sources Constrained to Field Measurements at Rural
North China Plain. Environ. Sci. Technol..

[95] Fu X., Wang T., Zhang L., Li Q., Wang Z., Xia M., Yun H., Wang W., Yu C., Yue D., Zhou Y., Zheng J., Han R. (2019). The significant
contribution
of HONO to secondary pollutants during a severe winter pollution event
in southern China. Atmos. Chem. Phys..

[96] Spataro F., Ianniello A. (2014). Sources of
atmospheric nitrous acid: State of the science,
current research needs, and future prospects. J. Air Waste Manage. Assoc..

