# Supplementary material for: Photochemical Modeling of Wintertime HO x Sources and Sinks in a Subarctic Urban Location
Source: ACS EST Air. 2026 Sep 12;3(10):2827–42. doi: 10.1021/acsestair.6c00254 (PMC13660409; doi:10.1021/acsestair.6c00254)
Supplement: Supplementary file 1 [file ea6c00254_si_001.pdf]

Photochemical modeling of wintertime HO_x sources and sinks in a sub-Arctic urban location – Supporting Information

Alicia Hoffman,[†] Rachel L. James,[‡,††] Dwayne E. Heard,[‡] Lisa Whalley,[‡,¶], Daniel Stone,[‡] Samuel Seldon,[‡] Jochen Stutz,[§] Jonas Kuhn,[§] Sol Cooperdock,[‖,§] Brice Temime-Roussel,[⊥] Amna Ijaz,[⊥] Barbara D'Anna,[⊥] Kathy S. Law,[#] Slimane Bekki,[#] Fangzhou Guo,[@,‡‡] James Flynn,[@] James M. St. Clair,[△] Meeta Cesler-Maloney,[∇] William R. Simpson,[∇] and Steve R. Arnold[*,†]

†*Institute for Climate and Atmospheric Science, School of Earth & Environment, University of Leeds, Leeds LS2 9JT, UK*
‡*School of Chemistry, University of Leeds, Leeds LS2 9JT, UK*
¶*National Centre for Atmospheric Sciences, University of Leeds, Leeds, LS2 9JT, UK*
§*UCLA Atmospheric & Oceanic Sciences, Los Angeles, CA 90095, USA*
‖*Woodwell Climate Research Center, Falmouth, MA 02540, USA*
⊥*Aix-Marseille Université, CNRS, LCE, Marseille, France*
#*LATMOS/IPSL, Sorbonne Université, UVSQ, CNRS, Paris, 75252, France*
@*Earth & Atmospheric Sciences, University of Houston, Houston, TX 77204, USA*
△*GESTAR-II, University of Maryland Baltimore County, Baltimore, MD 21250, USA*
∇*Geophysical Institute and Department of Chemistry and Biochemistry, University of Alaska Fairbanks, Fairbanks, AK 99775, USA*
††*now at: Department of Earth and Environmental Sciences, The University of Manchester, Manchester, UK*
‡‡*now at: Center for Atmospheric and Environmental Chemistry, Aerodyne Research Inc, Billerica, MA 01821, USA*

E-mail: S.Arnold@leeds.ac.uk

S1 Supplementary Figure

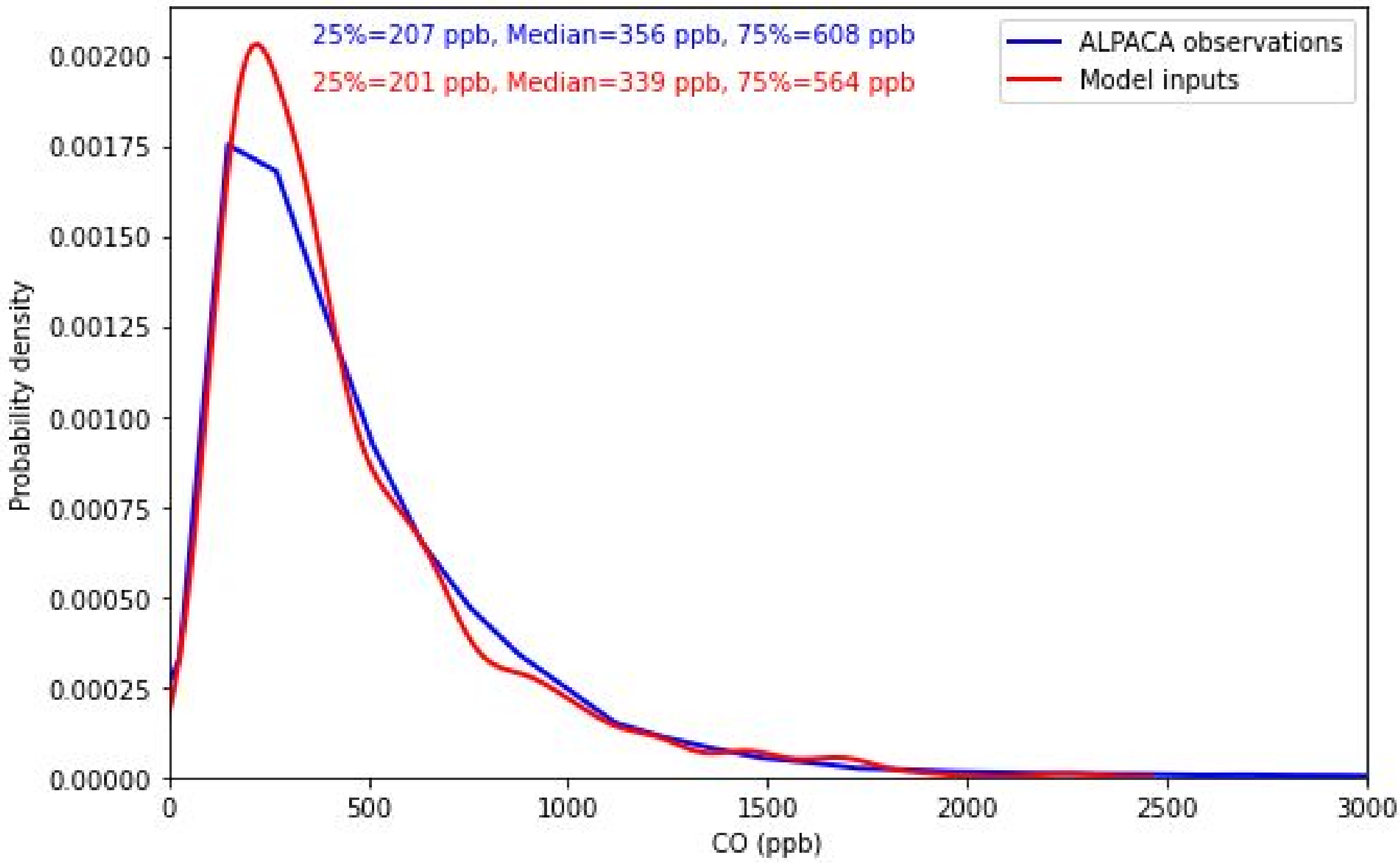

Figure S1: *Normalized kernel density estimate (KDE) distributions for ALPACA campaign period CO measurements (blue) and the subset of data points used for DSMACC box modelling (red). The 25th percentile, median, and 75th percentile values for each distribution are shown on the plot panel colored according to the legend. See main manuscript text for details of criteria for selection of valid model datapoints.*
